# Prediction of acute postoperative protein depletion risk in colon cancer using an in-context learning foundation model: a retrospective cohort study

**DOI:** 10.3389/fonc.2026.1857275

**Published:** 2026-07-08

**Authors:** Xinke Cao, Linrui Han, Xinquan Zan, Yinchao Zhang, Zhiqiang Tian, Wei Shen

**Affiliations:** The Affiliated Wuxi People’s Hospital of Nanjing Medical University, Wuxi People’s Hospital, Wuxi Medical Center, Nanjing Medical University, Wuxi, China

**Keywords:** acute postoperative protein depletion, colon cancer, perioperative nutrition, SHAP, TabICLv2

## Abstract

**Background:**

Acute postoperative protein depletion, including hypoalbuminaemia and hypoproteinaemia, frequently complicates colon cancer surgery and exacerbates adverse outcomes, yet early risk stratification remains challenging.

**Aims:**

To develop a predictive model utilising a tabular foundation model for acute postoperative protein depletion in colon cancer patients, alongside an interpretable clinical web tool.

**Methods:**

We retrospectively evaluated perioperative data from 812 colon cancer patients treated between 2020 and 2025. Following recursive feature elimination, eight traditional machine learning algorithms and the TabICLv2 tabular foundation model were trained. Discrimination, calibration, incremental risk stratification, and clinical utility were assessed using the area under the receiver operating characteristic curve (AUC), paired DeLong tests, calibration curves, Brier score, net reclassification improvement (NRI), integrated discrimination improvement (IDI), and decision curve analysis. SHapley Additive exPlanations (SHAP) were used to visualise feature contributions.

**Results:**

TabICLv2 achieved the numerically highest validation AUC among the evaluated algorithms using nine selected predictors, with an AUC of 0.766 (95% CI: 0.699–0.832) and a Brier score of 0.158. DeLong tests showed no statistically significant AUC differences between TabICLv2 and XGBoost or Random Forest. NRI/IDI analyses indicated improved event reclassification across comparator models, with significant total NRI and IDI improvement over Random Forest, whereas incremental improvement over Logistic Regression and XGBoost was limited. Sensitivity analyses using albumin-only and total-protein-only endpoints showed broadly consistent discrimination. SHAP analysis revealed age, prealbumin (PA), and globulin (GLO) as the leading contributors to model predictions. A web-based calculator was subsequently deployed to facilitate clinical translation. Conclusion: TabICLv2 integrates demographic, nutritional, and immunological profiles to predict acute postoperative protein depletion with moderate discrimination. The accompanying application may provide adjunctive individualised risk assessment, but prospective multicentre validation is required before routine clinical implementation.

## 1 Introduction

Colon cancer accounts for approximately 10% of global cancer diagnoses and remains one of the leading causes of cancer-related mortality (1, 2). Although advances in surgical techniques and perioperative management have improved clinical outcomes, postoperative complications remain common after colorectal surgery, with reported rates ranging from 26% to 35% (3). Among these complications, acute postoperative reductions in serum albumin and other circulating protein markers are frequently observed. Following major abdominal surgery, serum albumin can decrease rapidly, with an average reduction of approximately 33% within 48 hours (4). Given the physiological roles of albumin in maintaining oncotic pressure, ligand transport, and immune modulation, this acute decline is closely associated with adverse postoperative outcomes, including anastomotic leakage, surgical site infection, delayed recovery, and coagulopathy (5). Therefore, early identification of patients at risk of postoperative protein depletion may support timely nutritional assessment and perioperative optimisation.

Conventional clinical scoring systems and linear models may be insufficient to capture the complex, non-linear interactions among nutritional, inflammatory, coagulation-related, and tumour-associated biomarkers (6, 7). Machine learning (ML) methods provide a potential strategy for modelling such high-dimensional relationships; however, prediction models specifically targeting postoperative hypoalbuminaemia or broader acute postoperative protein depletion in colon cancer remain limited (8, 9, 11). Recent machine learning studies in colorectal cancer surgery have mainly focussed on outcomes such as postoperative length of stay, infectious complications, or anastomotic leakage rather than acute postoperative protein depletion. In addition, many existing models were developed in single-centre or relatively limited cohorts, incorporated incomplete perioperative protein-metabolism markers, and offered restricted interpretability for clinical decision-making (10). Beyond colorectal surgery, recent cancer research has increasingly emphasised the need for clinically translational and computationally grounded frameworks. Population-level burden studies in gastrointestinal malignancies highlight the continuing importance of risk stratification in digestive oncology (32), whereas patient-derived organoid systems have been proposed as translational platforms for individualised clinical decision-making and drug development (33). In parallel, studies integrating machine learning with single-cell analysis, network toxicology, and multi-omics approaches have illustrated how computational frameworks can link prediction with mechanistic interpretation (34, 36).

Tabular foundation models have recently emerged as a promising approach for structured clinical data analysis. Unlike conventional ML algorithms that often require dataset-specific hyperparameter optimisation, TabICLv2 applies a pre-trained tabular inference framework and uses labelled training samples as contextual examples when generating predictions for new observations (12). In this study, the term in-context learning refers to this tabular inference mechanism rather than language-based prompting. Although such models may reduce the need for extensive task-specific tuning, their performance and interpretability in predicting postoperative metabolic complications remain insufficiently evaluated. Therefore, this study aimed to develop and validate a risk stratification model for acute postoperative protein depletion in patients undergoing colon cancer surgery. We hypothesised that TabICLv2, combined with SHapley Additive exPlanations (SHAP), would provide competitive discrimination, acceptable calibration, and clinically interpretable risk attribution compared with conventional ML algorithms. We further sought to translate the model into an accessible web-based calculator to support individualised perioperative risk assessment and nutritional decision-making.

## 2 Materials and methods

### 2.1 Study design and ethical approval

**Figure 1:**
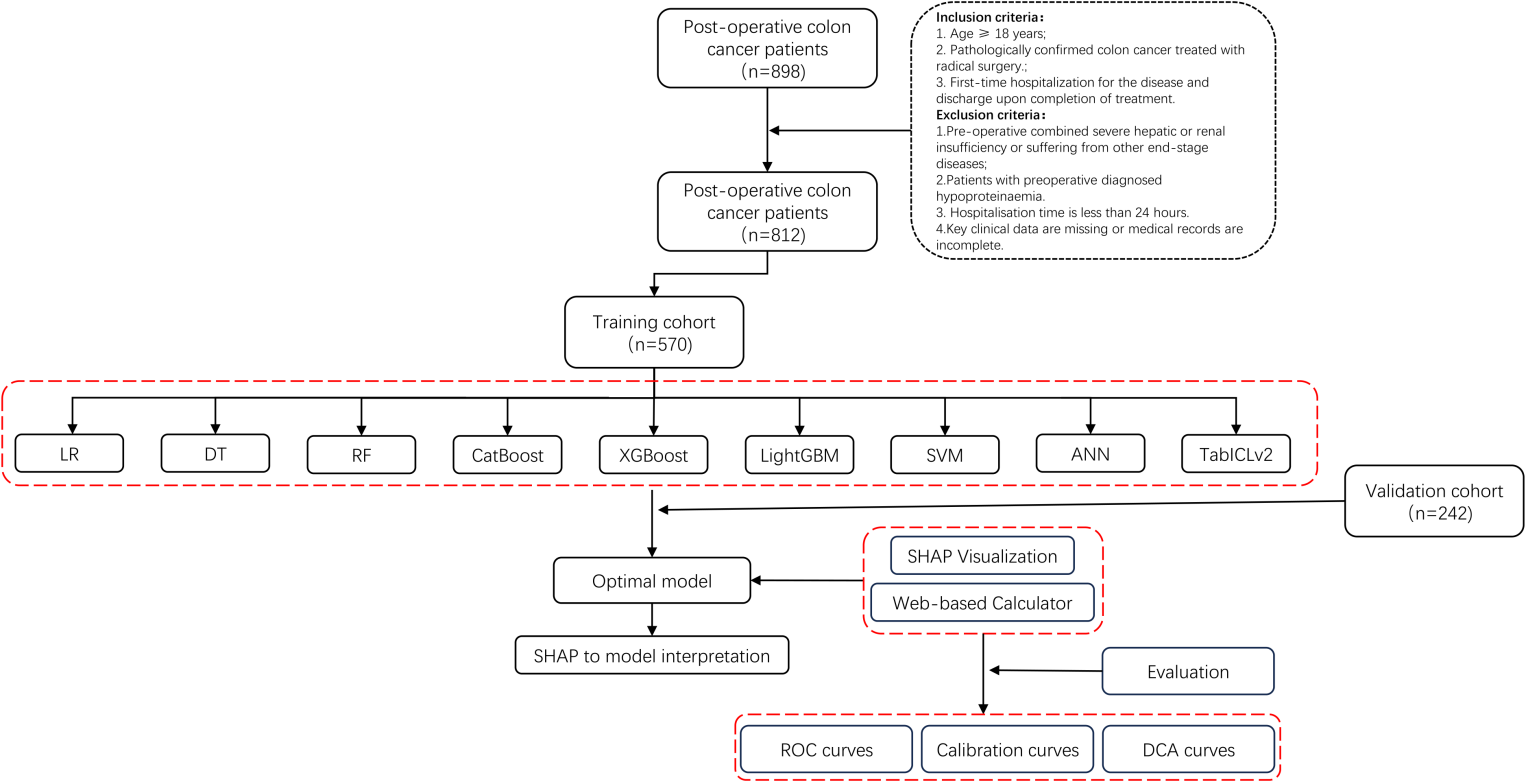
Flowchart of the study population selection process. The diagram illustrates the sequential application of inclusion and exclusion criteria to the initial cohort of 898 potentially eligible patients with colon cancer, yielding a final analytical cohort of 812 patients.

This single-centre retrospective cohort study analysed consecutive patients with colon cancer who underwent surgical resection at Wuxi People’s Hospital between January 2020 and December 2025. An initial query of the institutional electronic medical record (EMR) system identified 898 potentially eligible patients. Following the application of predefined selection criteria, a final cohort of 812 patients was established for model development and validation (**Figure 1**).

The study protocol was approved by the Institutional Review Board of Wuxi People’s Hospital before data analysis (approval number: KY26124). The study was conducted in accordance with the Declaration of Helsinki. Given the observational nature of this research and the use of anonymised retrospective data, the requirement for written informed consent was waived by the committee.

### 2.2 Study population

Patients were included if they were: (1) aged ≥ 18 years; (2) diagnosed with histopathologically confirmed primary colon cancer; and (3) undergoing their first elective radical resection.

Patients were excluded based on the following criteria: (1) baseline preoperative hypoalbuminaemia; (2) pre-existing severe hepatic or renal dysfunction, or end-stage comorbidities capable of altering baseline protein metabolism; (3) a postoperative hospital stay of less than 24 hours; and (4) unavailable postoperative albumin or total protein measurements required for endpoint determination.

### 2.3 Data extraction and outcome definition

Clinical data were extracted from the “Yidu Cloud” EMR system, encompassing preoperative evaluations conducted within one week prior to surgery. Extracted variables included patient demographics, vital signs, comorbidities, and comprehensive laboratory indices, including complete blood counts, biochemical parameters, coagulation profiles, and tumour markers (**Table 1**). To prevent information loss during model training, continuous laboratory variables were retained as raw numerical values rather than being categorised.

**Table 1:** Comparison of baseline clinical and laboratory characteristics of the study population.

Characteristics*^a^*	Yes (n=596)	No (n=216)	*P* value*^b^*	SMD*^c^*
Gender, n (%)			0.612	0.040
Male	333 (55.9%)	125 (57.9%)		
Female	263 (44.1%)	91 (42.1%)		
Hypertension, n (%)			0.328	0.078
No	333 (55.9%)	129 (59.7%)		
Yes	263 (44.1%)	87 (40.3%)		
CHD, n (%)			0.448	0.063
No	576 (96.6%)	211 (97.7%)		
Yes	20 (3.4%)	5 (2.3%)		
Diabetes, n (%)			0.201	0.104
No	509 (85.4%)	192 (88.9%)		
Yes	87 (14.6%)	24 (11.1%)		
Age (months), median (IQR)	834.5 (729.75, 915.25)	754 (665, 839.5)	<0.001	**0.541**
Temperature, median (IQR)	36.6 (36.5, 36.8)	36.6 (36.5, 36.8)	0.793	0.019
Pulse, median (IQR)	78 (74, 86)	78 (74, 84)	0.262	0.120
Heart rate, median (IQR)	78 (74, 87)	78 (74, 84)	0.228	0.108
Diastolic blood pressure, median (IQR)	78 (70, 83)	79.5 (73, 83)	0.297	0.063
Systolic blood pressure, median (IQR)	130 (120, 142)	128 (120, 140)	0.642	0.041
Mono%, median (IQR)	7.6 (6.275, 9)	7.2 (6.175, 8.5)	0.060	0.185
WBC, median (IQR)	6.64 (5.3475, 8.2925)	6.615 (5.425, 8.5325)	0.796	0.009
RBC, mean ± SD	4.2328 ± 0.64027	4.387 ± 0.64225	0.003	**0.241**
MCV, median (IQR)	88.3 (81.675, 92.3)	89.4 (84.15, 92.225)	0.165	0.111
MCHC, median (IQR)	322 (309, 333)	328 (316, 336)	0.001	**0.216**
MCH, median (IQR)	29 (25.075, 30.5)	29.4 (27.1, 30.9)	0.022	0.166
RDW-CV, median (IQR)	13.2 (12.6, 15)	12.85 (12.4, 13.925)	<0.001	**0.245**
Lymph%, median (IQR)	23.25 (16.9, 30.625)	26.3 (21.5, 32.6)	<0.001	**0.355**
Lymphocyte count, median (IQR)	1.58 (1.19, 1.91)	1.73 (1.3975, 2.1725)	<0.001	**0.411**
Mono, median (IQR)	0.5 (0.39, 0.65)	0.475 (0.38, 0.61)	0.096	0.180
Neut%, mean ± SD	66.074 ± 10.908	63.311 ± 9.4342	<0.001	**0.271**
Neut, median (IQR)	4.39 (3.1675, 5.8725)	4.025 (3.09, 5.5875)	0.324	0.085
Hct, median (IQR)	37.5 (31.675, 41.7)	39.1 (35.1, 43)	0.001	**0.252**
Eos%, median (IQR)	1.4 (0.6, 2.7)	1.5 (0.8, 2.5)	0.517	0.016
Baso%, median (IQR)	0.3 (0.2, 0.5)	0.4 (0.275, 0.6)	0.006	**0.216**
Eos, median (IQR)	0.09 (0.04, 0.17)	0.1 (0.05, 0.1525)	0.585	0.000
Baso, median (IQR)	0.02 (0.01, 0.04)	0.03 (0.02, 0.04)	0.013	0.188
Hb, median (IQR)	121 (96, 138)	129 (112.75, 142.25)	<0.001	**0.262**
PLT, median (IQR)	256.5 (202, 322)	250.5 (200.25, 314)	0.521	0.054
MPV, median (IQR)	10.3 (9.7, 11.1)	10.3 (9.7, 11.1)	0.657	0.051
PDW, median (IQR)	11.7 (10.4, 13.4)	11.7 (10.4, 13.4)	0.537	0.066
Platelet volume, median (IQR)	0.27 (0.21, 0.33)	0.26 (0.21, 0.32)	0.514	0.051
ALT, median (IQR)	13 (9.7, 19.125)	15.35 (11.2, 22.05)	<0.001	0.194
AST, median (IQR)	14 (7, 21.25)	11 (5, 20)	0.011	0.183
GGT, median (IQR)	20 (15, 31)	20 (16, 31.25)	0.222	0.083
AST/ALT, median (IQR)	1.44 (1.12, 1.79)	1.265 (0.9875, 1.5925)	<0.001	**0.346**
TBIL, median (IQR)	11.3 (8.7, 15.2)	11.6 (9.6, 15.5)	0.307	0.017
DBIL, median (IQR)	3.5 (2.7, 4.8)	3.4 (2.7, 4.6)	0.511	0.080
ALB, mean ± SD	38.419 ± 4.8275	41.057 ± 4.2181	<0.001	**0.582**
GLO, median (IQR)	28.3 (25.2, 31.9)	29.2 (26.4, 32.025)	0.011	0.168
TP, median (IQR)	66.7 (61.8, 72.325)	70.05 (65.6, 75.55)	<0.001	**0.468**
ALB/GLO, median (IQR)	1.365 (1.19, 1.53)	1.395 (1.27, 1.5625)	0.070	0.154
PA, median (IQR)	185 (139.3, 230.25)	232.7 (191.82, 274.12)	<0.001	**0.693**
TBA, median (IQR)	3 (1.7, 5)	2.6 (1.5, 4.025)	0.004	**0.312**
Crea, median (IQR)	68.8 (59.475, 81.7)	69.75 (59.1, 78.825)	0.492	0.104
Urea, median (IQR)	5.1 (4.1, 6.4)	5 (4.2, 5.9)	0.322	0.103
UA, median (IQR)	292.2 (240.03, 358.1)	304.45 (269.02, 363.57)	0.007	**0.236**
Urea/Crea, median (IQR)	0.073 (0.058, 0.091)	0.073 (0.05875, 0.087)	0.728	0.033
K+, median (IQR)	3.93 (3.63, 4.19)	3.895 (3.6775, 4.1525)	0.964	0.024
Na+, median (IQR)	140.5 (138.8, 141.9)	140.8 (139.7, 142.2)	0.005	**0.274**
Cl-, median (IQR)	105 (102.97, 106.93)	105.1 (103.57, 106.93)	0.291	0.149
Ca, median (IQR)	2.23 (2.14, 2.31)	2.28 (2.22, 2.4)	<0.001	**0.597**
P, median (IQR)	1.12 (1, 1.27)	1.16 (1.04, 1.29)	0.024	0.157
Mg2+, median (IQR)	0.87 (0.83, 0.92)	0.88 (0.84, 0.93)	0.013	**0.225**
Glu, median (IQR)	5.28 (4.72, 6.4525)	5.26 (4.67, 6.1375)	0.286	0.166
ALP, median (IQR)	81 (69, 98)	78 (66, 94)	0.059	**0.227**
ADA, median (IQR)	8.95 (7, 11.2)	8.8 (6.675, 11.1)	0.440	0.080
ChE, median (IQR)	6276.5 (5123.8, 7498.8)	7402.5 (6319.2, 8488.5)	<0.001	**0.628**
Cys-C, median (IQR)	0.83 (0.7, 0.98)	0.805 (0.71, 0.91)	0.133	0.175
CO2CP, mean ± SD	26.801 ± 2.5665	27.113 ± 2.4132	0.119	0.126
eGFR, median (IQR)	87.8 (77.2, 96.725)	92.95 (85.35, 99.625)	<0.001	**0.374**
Osm, median (IQR)	281 (278, 285)	282 (279, 284)	0.098	0.134
AG, median (IQR)	8.25 (7.075, 10)	8.5 (7.3, 10.1)	0.679	0.046
PT-INR, median (IQR)	1.06 (1.01, 1.12)	1.05 (1, 1.09)	0.013	**0.239**
PT, median (IQR)	12 (11.4, 12.6)	11.8 (11.2, 12.3)	<0.001	**0.295**
TT, median (IQR)	20.35 (19, 21.7)	20.35 (19, 22.025)	0.482	0.080
ATIII:A, mean ± SD	91.077 ± 15.351	96.824 ± 14.298	<0.001	**0.387**
APTT, mean ± SD	29.756 ± 3.0929	30.081 ± 2.9645	0.182	0.107
Fbg, median (IQR)	3.245 (2.7875, 3.78)	3.085 (2.58, 3.4525)	<0.001	**0.337**
FDP, median (IQR)	1.665 (0.89, 3.8275)	1.155 (0.72, 1.78)	<0.001	**0.426**
D-Dimer, median (IQR)	214.5 (114.75, 455.25)	142.5 (80, 238)	<0.001	**0.400**
AFP, median (IQR)	2.61 (1.915, 3.435)	2.64 (2.04, 3.4675)	0.913	0.133
CEA, median (IQR)	3.745 (1.95, 11.865)	2.505 (1.4675, 6.8625)	<0.001	0.176
CA125, median (IQR)	10.6 (6.7, 20.35)	8.8 (6.1, 12.375)	<0.001	**0.278**
CA199, median (IQR)	13.55 (7.275, 35.35)	11.4 (6.4, 25.525)	0.019	0.117
LDH, median (IQR)	176 (154.75, 210)	174.5 (155, 201)	0.357	0.129

Yes and No refer to the presence or absence of acute postoperative protein depletion according to the composite endpoint. Variables with missingness greater than 30% were excluded from the main baseline comparison table and model development, and are summarised separately in **Supplementary Table 4**.IQR, interquartile range; SD, standard deviation; SMD, standardised mean difference; CHD, coronary heart disease; WBC, white blood cell count; RBC, red blood cell count; MCV, mean corpuscular volume; MCH, mean corpuscular haemoglobin; MCHC, mean corpuscular haemoglobin concentration; RDW-CV, red blood cell distribution width-coefficient of variation; Lymph, lymphocyte count; Lymph%, lymphocyte percentage; Mono, monocyte; Neut, neutrophil; Eos, eosinophil; Baso, basophil; Hb, haemoglobin; Hct, haematocrit; PLT, platelet count; MPV, mean platelet volume; PDW, platelet distribution width; ALT, alanine aminotransferase; AST, aspartate aminotransferase; GGT, gamma-glutamyl transferase; TBIL, total bilirubin; DBIL, direct bilirubin; ALB, albumin; GLO, globulin; TP, total protein; PA, prealbumin; TBA, total bile acid; Crea, creatinine; UA, uric acid; K+, potassium; Na+, sodium; Cl-, chloride; Ca, calcium; P, phosphorus; Mg2+, magnesium; Glu, glucose; ALP, alkaline phosphatase; ADA, adenosine deaminase; ChE, cholinesterase; Cys-C, cystatin C; CO2CP, carbon dioxide combining power; eGFR, estimated glomerular filtration rate; Osm, osmolality; AG, anion gap; PT, prothrombin time; PT-INR, prothrombin time-international normalised ratio; TT, thrombin time; ATIII:A, antithrombin III activity; APTT, activated partial thromboplastin time; Fbg, fibrinogen; FDP, fibrin degradation products; D-Dimer, D-dimer; AFP, alpha-foetoprotein; CEA, carcinoembryonic antigen; CA125, carbohydrate antigen 125; CA19-9, carbohydrate antigen 19-9; LDH, lactate dehydrogenase.

*^a^*Data are presented as mean ± SD for normally distributed continuous variables, median (IQR) for non-normally distributed continuous variables, and frequencies (percentages) for categorical variables. *^b^*Group comparisons were performed using the independent samples t-test, Mann-Whitney U test, Pearson’s Chi-square test, or Fisher’s exact test, as appropriate. A two-sided *P <* 0.05 was considered statistically significant. *^c^*SMD represents the absolute standardised mean difference between patients with and without acute postoperative protein depletion. SMD values *>* 0.20 were considered potentially clinically meaningful and are shown in bold.

The primary endpoint was acute postoperative protein depletion, defined as either a serum albumin nadir of *<* 35 g/L or a serum total protein nadir of *<* 60 g/L recorded during the first postoperative week. Serum albumin *<* 35 g/L has been widely used to define hypoalbuminaemia in colon cancer and perioperative surgical studies (16, 17, 40). Serum total protein *<* 60 g/L was selected because this value approximates the lower boundary of commonly reported adult laboratory reference ranges for circulating total protein and has been used to indicate hypoproteinaemia or reduced circulating protein reserve (41). This composite endpoint was intended to capture early postoperative protein depletion rather than isolated hypoalbuminaemia alone, because postoperative protein loss may be reflected by reductions in either albumin or total circulating protein. Because this composite endpoint is not a universally guideline-defined diagnostic standard, albumin-only and total-protein-only sensitivity analyses were prespecified and performed separately to assess the robustness of the findings.

### 2.4 Statistical analysis and pre-processing

Descriptive statistics and pre-processing were conducted using R (version 4.2.3) and Python (version 3.10.11; scikit-learn and tabicl libraries). Variables with *>* 30% missingness were excluded, whereas variables with ≤ 30% missing data were imputed using Multiple Imputation by Chained Equations (MICE). The extent of missingness for candidate variables was summarised in **Supplementary Table 4**. Variables with missingness greater than 30% were excluded from model development and from the main baseline comparison table as a predefined pragmatic data-quality threshold. This threshold was used to reduce instability from variables with excessive missingness rather than as an absolute statistical rule, because the proportion of missingness alone should not determine whether multiple imputation is appropriate (42). Variables with missingness of 30% or less were retained and imputed using MICE (43). Five imputed datasets were generated with 10 iterations per dataset. A complete-case sensitivity analysis was additionally performed for the final TabICLv2 model among patients with complete data for the nine selected predictors and the primary outcome. The model was retrained and evaluated without imputation to assess the robustness of the main MICE-based analysis. Normality of continuous variables was evaluated using the Kolmogorov-Smirnov test. Normally distributed data were expressed as mean ± standard deviation (SD) and compared using Student’s t-test, whereas non-normally distributed data were reported as median (interquartile range, IQR) and compared using the Mann-Whitney U test. Categorical variables were presented as frequencies (percentages) and analysed with Pearson’s Chi-square or Fisher’s exact test. Exact *P* values were reported where applicable, with two-sided *P <* 0.05 considered statistically significant. Standardised mean differences (SMDs) were calculated to assess the magnitude of between-group differences. An absolute SMD *>* 0.20 was considered potentially clinically meaningful and was highlighted in **Table 1**. Univariable logistic regression was further performed to provide an exploratory assessment of baseline variables associated with acute postoperative protein depletion. Each baseline variable listed in **Table 1** was entered separately into a logistic regression model, with acute postoperative protein depletion as the dependent variable. Odds ratios (ORs), 95% confidence intervals (CIs), and two-sided *P* values were reported. These univariable analyses were conducted for descriptive and exploratory purposes and were not used as the basis for machine-learning feature selection.

### 2.5 Statistical formulae

To enhance methodological transparency, the main statistical procedures and evaluation metrics were expressed using standard formulae (43, 46, 47).

For MICE, missing values in each incomplete variable $X_{j}$ were iteratively imputed from a conditional model based on the remaining variables:


$$X_{j}^{mis}∼P(X_{j}|X_{-j},\theta_{j}),\;j=1,\ldots ,p.$$

This procedure cycles through incomplete variables and generates multiple plausible completed datasets, thereby accounting for uncertainty due to missingness.

The Pearson correlation coefficient was calculated as:


$$r_{xy}=\frac{\sum_{i=1}^{n}(x_{i}-\bar{x})(y_{i}-\bar{y})}{\sqrt{\sum_{i=1}^{n}{\left(x_{i}-\bar{x}\right)}^{2}\sum_{i=1}^{n}{\left(y_{i}-\bar{y}\right)}^{2}}}.$$

This coefficient quantifies the strength and direction of the linear association between two continuous variables.

Univariable logistic regression was expressed as:


$$\log\;(\frac{p_{i}}{1-p_{i}})=\beta_{0}+\beta_{1}X_{i},$$

where *p_i_* denotes the probability of acute postoperative protein depletion for patient *i*. The exponentiated coefficient, exp (β_1_), represents the odds ratio associated with a one-unit increase in the predictor.

The Brier score was calculated as:


$$BS=\frac{1}{n}\sum_{i=1}^{n}{\left({\hat{p}}_{i}-y_{i}\right)}^{2},$$

where ${\hat{p}}_{i}$ is the predicted probability and $y_{i}$is the observed binary outcome. Lower Brier scores indicate better overall probabilistic accuracy and calibration.

Continuous net reclassification improvement (NRI) was calculated as:


$$NRI=[P_{\text{up}|\text{event}}-P_{\text{down}|\text{event}}]+[P_{\text{down}|\text{nonevent}}-P_{\text{up}|\text{nonevent}}].$$

NRI evaluates whether the new model assigns higher predicted risks to event cases and lower predicted risks to non-event cases compared with a reference model.

Integrated discrimination improvement (IDI) was calculated as:


$$IDI=({\bar{p}}_{\text{event},\text{new}}-{\bar{p}}_{\text{event},\text{old}})-({\bar{p}}_{\text{nonevent},\text{new}}-{\bar{p}}_{\text{nonevent},\text{old}}).$$

IDI quantifies the improvement in separation between predicted risks for event and non-event patients after replacing the reference model with the new model.

### 2.6 Sample size considerations

As this was a single-centre retrospective cohort study based on consecutive eligible patients, the sample size was determined by the number of available cases during the predefined study period rather than by an *a priori* power calculation. The final cohort of 812 patients was established before the 7:3 training-validation split. To provide a *post-hoc* justification, 596 outcome events were observed in the overall cohort. Although more than 60 baseline variables were initially considered, dimensionality reduction was performed using correlation filtering followed by recursive feature elimination within the training cohort. The final prediction model included nine predictors, resulting in an event-to-final-predictor ratio of 66.2, which exceeded the commonly cited minimum of 10 events per predictor for prediction modelling. Nevertheless, because the initial candidate feature pool was relatively large and feature selection was data-driven, residual overfitting risk cannot be completely excluded.

### 2.7 Feature selection and model construction

To mitigate multicollinearity, highly correlated variables (Pearson |*r*| *>* 0.70) were selectively removed based on clinical relevance, data completeness, and relevance to perioperative protein metabolism. The dataset was randomly partitioned into a training set (70%) and a validation set (30%) using stratified sampling according to the primary composite outcome. A 7:3 split was selected to preserve most observations for model training while retaining an independent validation subset of sufficient size for internal performance assessment. This approach provides a practical balance between model development stability and validation precision; in contrast, 5:5 or 6:4 splits would further reduce the number of training observations available for feature selection, cross-validation, and model fitting in this single-centre cohort (44, 45). Stratified sampling was applied to maintain a similar outcome-event distribution in both subsets.

Feature selection was performed exclusively within the training cohort to avoid information leakage. Recursive Feature Elimination (RFE) was conducted using five-fold cross-validation. Candidate feature subsets were iteratively evaluated according to cross-validated model performance, and the final feature set was determined at the point where cross-validated performance reached its optimal or near-optimal plateau. When adjacent feature subsets showed comparable performance, the smaller subset was preferred to reduce model complexity. This process identified nine core predictors, which were fixed before validation-set evaluation.

Subsequently, eight traditional machine learning classifiers (Logistic Regression, SVM, Decision Tree, Random Forest, ANN, LightGBM, CatBoost, and XGBoost) were trained. For these conventional models, hyperparameter optimisation was performed using the Optuna framework with five-fold cross-validation in the training cohort to maximise AUC. The main hyperparameter search spaces and final optimal configurations are provided in **Supplementary Table 1**.

TabICLv2 was implemented as a pre-trained tabular foundation model using its tabular in-context inference mechanism (12). In this setting, labelled training samples served as contextual examples for generating predictions on validation samples, rather than requiring language-based prompting. No dataset-specific hyperparameter search was performed for TabICLv2, and the same nine predictors were used as inputs for all models.

### 2.8 Model evaluation and interpretability

Model discrimination was quantified using receiver operating characteristic (ROC) curves and the AUC. To formally compare the discrimination of TabICLv2 with the strongest conventional models, paired DeLong tests were performed to compare validation AUCs between TabICLv2 and XGBoost, and between TabICLv2 and Random Forest. AUC differences were calculated as TabICLv2 minus the comparator model, and two-sided *P* values with 95% confidence intervals (CIs) for the differences were reported. To further assess incremental risk stratification beyond conventional models, continuous/category-free net reclassification improvement (NRI) and integrated discrimination improvement (IDI) were calculated in the validation cohort. TabICLv2 was compared with Logistic Regression, Random Forest, and XGBoost using predicted probabilities rather than binary classifications. Positive NRI or IDI values indicated improved reclassification or discrimination by TabICLv2 relative to the comparator model. Confidence intervals and two-sided *P* values were estimated using bootstrap resampling with 2000 iterations.

Training-validation AUC differences were further calculated to assess potential apparent optimism. Apparent training AUC was obtained by applying the final fitted model to the training cohort, whereas validation AUC was calculated in the independent validation cohort. AUC difference was defined as apparent training-cohort AUC minus validation-cohort AUC. Confidence intervals and two-sided *P* values for the AUC differences were estimated using bootstrap resampling with 2000 iterations, with separate resampling of the training and validation cohorts.

The Youden Index dictated the optimal classification threshold, from which sensitivity, specificity, accuracy, positive predictive value (PPV), negative predictive value (NPV), F1 score, and Cohen’s Kappa were derived.

To address the potential heterogeneity introduced by the composite endpoint, sensitivity analyses were performed using two alternative outcome definitions: albumin-only hypoalbuminaemia, defined as a postoperative serum albumin nadir of *<* 35 g/L during the first postoperative week, and total-protein-only hypoproteinaemia, defined as a postoperative serum total protein nadir of *<* 60 g/L during the same period. For comparability with the primary analysis, the same nine predictors and a consistent 7:3 training-validation modelling framework were used, while only the outcome definition was changed. The TabICLv2 model was retrained separately for each alternative endpoint, and model performance was assessed using AUC, optimal-threshold-based classification metrics, calibration curves, and Brier scores.

To elucidate the decision-making rationale of the TabICLv2 model, the SHapley Additive exPlanations (SHAP) framework was integrated. Global feature importance and directional associations were visualised using summary beeswarm plots, while localised waterfall and force plots were generated to demonstrate individualised risk attribution.

To further explore potential combined effects among core predictors, exploratory SHAP-based pairwise interaction visualisations were generated for clinically relevant feature pairs, including PA–age, PA–GLO, PA–Lymph%, age–GLO, PA–ChE, and GLO–lymphocyte count. Each plot displayed the SHAP value of the index feature against its observed value, with point colour representing the paired clinical variable. LOWESS-smoothed curves were added to visualise overall trends. Because TabICLv2 is not a tree-ensemble model, exact TreeSHAP interaction values were not calculated; instead, these plots were used for exploratory visual assessment of potential pairwise interaction patterns. Age was displayed in years after conversion from the original month-based age variable.

## 3 Results

### 3.1 Patient baseline characteristics

The final analysis comprised 812 patients who underwent elective radical resection for colon cancer. Within this cohort, 596 patients (73.4%) developed acute postoperative protein depletion according to the composite endpoint, whereas 216 patients (26.6%) did not meet either protein-depletion criterion and served as controls. Baseline characteristics were broadly comparable between the two groups with respect to sex distribution, vital signs, and major comorbidities, including hypertension, coronary heart disease, and diabetes mellitus. However, several renal and electrolyte-related indices also differed between groups, including eGFR, UA, Na+, Ca, P, and Mg2+.

Significant inter-group variations were also observed across multiple haematological and biochemical indices. Patients in the protein-depletion cohort were significantly older (median age: 69.5 vs. 62.8 years, *P <* 0.001). They presented with lower preoperative levels of nutritional and hepatic synthesis markers, including prealbumin (PA), cholinesterase (ChE), total protein (TP), albumin (ALB), and haemoglobin (Hb) (all *P <* 0.05). Furthermore, immunological parameters, notably lymphocyte indices, were significantly depressed in this group.

Conversely, the protein-depletion group exhibited elevated baseline markers indicative of immune-inflammatory alteration and a hypercoagulable state, including neutrophil percentage (Neut%), fibrin degradation products (FDP), D-dimer, and prothrombin time (PT). Circulating tumour markers, including carcinoembryonic antigen (CEA) and carbohydrate antigen 125 (CA125), were also significantly higher in these patients (**Table 1**). SMD analysis further indicated potentially clinically meaningful between-group differences (absolute SMD *>* 0.20) for several nutritional, hepatic synthetic, coagulation-related, immune-inflammatory, and renal/electrolyte variables, with the largest differences observed for PA, ChE, Ca, ALB, age, TP, FDP, lymphocyte count, and D-dimer (**Table 1**). Univariable logistic regression further supported the associations between baseline clinical variables and acute postoperative protein depletion. Lower PA, ChE, Ca, ALB, TP, lymphocyte count, Lymph%, ATIII:A, and eGFR were associated with increased risk, whereas older age and higher FDP, D-dimer, AST/ALT, Fbg, PT, TBA, Neut%, and CA125 were positively associated with the outcome. These findings were broadly consistent with the subsequent feature-selection results and suggested that acute postoperative protein depletion was related to the combined effects of impaired nutritional reserve, hepatic synthetic dysfunction, immune-inflammatory alteration, coagulation activation, and electrolyte imbalance (**Supplementary Table 6**).

### 3.2 Feature extraction and model construction

To mitigate multicollinearity, Pearson correlation analysis was performed across all continuous baseline variables (**Supplementary Figure 1**). Using a threshold of |*r*| ≥ 0.70, highly correlated feature pairs were pruned based on clinical relevance and data completeness (e.g., retaining neutrophil percentage over absolute count, and pulse rate over redundant heart rate). This filtering process yielded a reduced set of independent candidate variables for subsequent RFE.

Subsequently, Recursive Feature Elimination (RFE) identified an optimal, non-redundant subset of nine core predictors (**Supplementary Figure 2**). These nine variables served as the standard input matrix for training both the eight traditional machine learning algorithms and the TabICLv2 foundation model.

### 3.3 Performance comparison and model selection

Hyperparameters for the traditional machine learning algorithms were optimised using the Optuna framework with five-fold cross-validation (**Supplementary Table 1**). In contrast, TabICLv2 used its pre-trained tabular in-context inference mechanism without dataset-specific hyperparameter optimisation.

**Figure 2:**
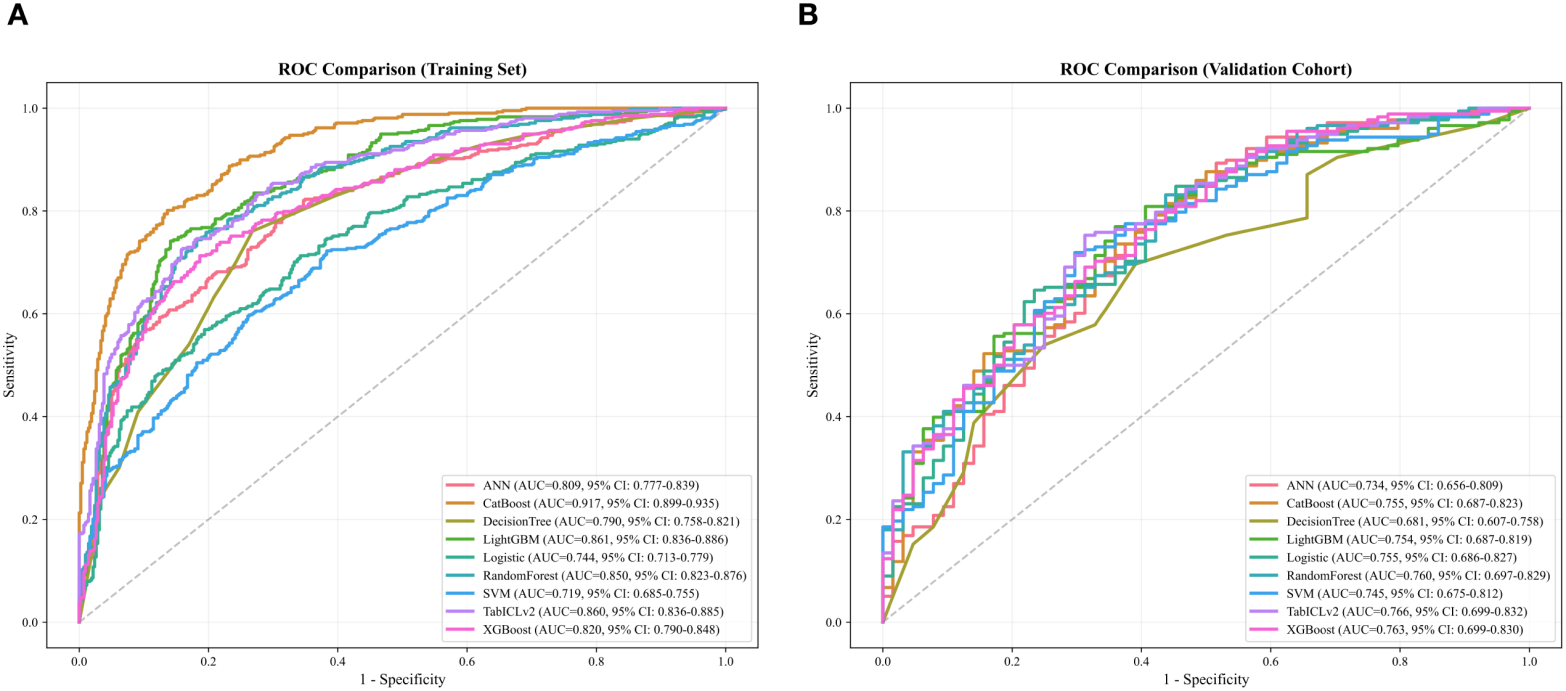
Receiver operating characteristic (ROC) curves of the evaluated predictive models. **(A)** Performance in the training cohort. **(B)** Performance in the validation cohort. Abbreviations: AUC, area under the curve; TabICLv2, Tabular In-Context Learning Version 2; XGBoost, Extreme Gradient Boosting; RF, Random Forest; SVM, Support Vector Machine; LR, Logistic Regression; DT, Decision Tree; ANN, Artificial Neural Network; LightGBM, Light Gradient Boosting Machine.

Performance metrics and receiver operating characteristic (ROC) curves are presented in **Table 2** and **Figure 2**. Training-validation AUC differences were further assessed to quantify potential apparent optimism. Significant AUC decreases were observed for Decision Tree, Random Forest, LightGBM, CatBoost, and TabICLv2, whereas Logistic Regression, SVM, ANN, and XGBoost did not show statistically significant AUC decreases. The largest AUC difference was observed for CatBoost (AUC difference = 0.162, 95% CI: 0.121–0.265; *P <* 0.001). These findings indicate that validation-cohort performance should be regarded as the primary basis for model comparison (**Supplementary Table 8**).

**Table 2:** Performance comparison of machine learning models at optimal thresholds in the validation cohort.

Model*^c^*	Optimal threshold	AUC (95% CI)	Accuracy*^b^*	PPV*^b^*	Sensitivity*^b^*	Specificity*^b^*	F1 Score*^b^*	Cohen’s Kappa*^b^*	NPV
ANN	0.591	0.734 (0.656–0.809)	0.785	0.828	0.893	0.484	0.859	0.406	0.620
CatBoost	0.659	0.755 (0.687–0.823)	0.748	0.838	0.815	0.563	0.826	0.368	0.522
Decision Tree	0.743	0.681 (0.607–0.758)	0.674	0.832	0.697	0.609	0.758	0.267	0.419
LightGBM	0.699	0.754 (0.687–0.819)	0.736	0.856	0.770	0.641	0.811	0.376	0.500
Logistic	0.730	0.755 (0.686–0.827)	0.678	0.885	0.646	0.766	0.747	0.332	0.438
Random Forest	0.646	0.760 (0.697–0.829)	0.760	0.841	0.831	0.563	0.836	0.390	0.545
SVM	0.713	0.745 (0.675–0.812)	0.715	0.871	0.719	0.703	0.788	0.366	0.474
XGBoost	0.723	0.763 (0.699–0.830)	0.690	0.860	0.691	0.688	0.766	0.322	0.444
**TabICLv2*^a^***	**0.689**	**0.766 (0.699–0.832)**	**0.736**	**0.870**	**0.753**	**0.688**	**0.807**	**0.393**	**0.500**

AUC, area under the receiver operating characteristic curve; PPV, positive predictive value; NPV, negative predictive value.

*^a^*Bold values indicate the final selected model, which achieved the highest validation AUC point estimate.

*^b^*Classification metrics, including accuracy, positive predictive value (PPV), sensitivity, specificity, F1 score, and Cohen’s kappa, were calculated at the optimal threshold determined by the Youden Index for each algorithm. *^c^*All metrics are derived from the independent validation cohort.

In the validation cohort, TabICLv2 achieved the highest AUC point estimate among the evaluated algorithms, with an AUC of 0.766 (95% CI: 0.699–0.832). This was comparable to the performance of XGBoost (AUC = 0.763), Random Forest (AUC = 0.760), and Logistic Regression (AUC = 0.755), while requiring no dataset-specific hyperparameter optimisation. Pairwise DeLong tests showed that the validation AUC difference between TabICLv2 and XGBoost was small and not statistically significant (AUC difference = 0.003, 95% CI: -0.030 to 0.037; two-sided *P* = 0.854). Similarly, the AUC difference between TabICLv2 and Random Forest was not statistically significant (AUC difference = 0.006, 95% CI: -0.024 to 0.035; two-sided *P* = 0.712). Therefore, TabICLv2 was interpreted as achieving the numerically highest validation AUC point estimate with comparable discrimination to the strongest conventional models, rather than statistically superior discriminative capacity. The full DeLong test results are provided in **Supplementary Table 3**.

Continuous NRI and IDI analyses were further performed to evaluate incremental risk stratification in the validation cohort. Compared with Logistic Regression, TabICLv2 significantly improved event reclassification (NRI event = 0.315, 95% CI: 0.169–0.458; *P <* 0.001), although the total NRI and IDI were not statistically significant. Compared with Random Forest, TabICLv2 showed significant improvement in total NRI (0.493, 95% CI: 0.228–0.782; *P <* 0.001) and IDI (0.035, 95% CI: 0.017–0.053; *P <* 0.001). Compared with XGBoost, TabICLv2 significantly improved event reclassification (NRI event = 0.303, 95%CI: 0.170–0.442; *P <* 0.001), whereas the total NRI and IDI were not statistically significant. These findings suggest that TabICLv2 provided improved event-risk ranking and modest incremental risk-stratification benefit, particularly relative to Random Forest, while its overall improvement over Logistic Regression and XGBoost remained limited (**Supplementary Table 7**).

Using the Youden Index, the optimal probability threshold was determined to be 0.689. At this cut-off, TabICLv2 achieved a sensitivity of 0.753, a specificity of 0.688, and an overall accuracy of 0.736. The F1 score was 0.807, and Cohen’s Kappa was 0.393.

### 3.4 Model calibration and clinical benefit

**Figure 3:**
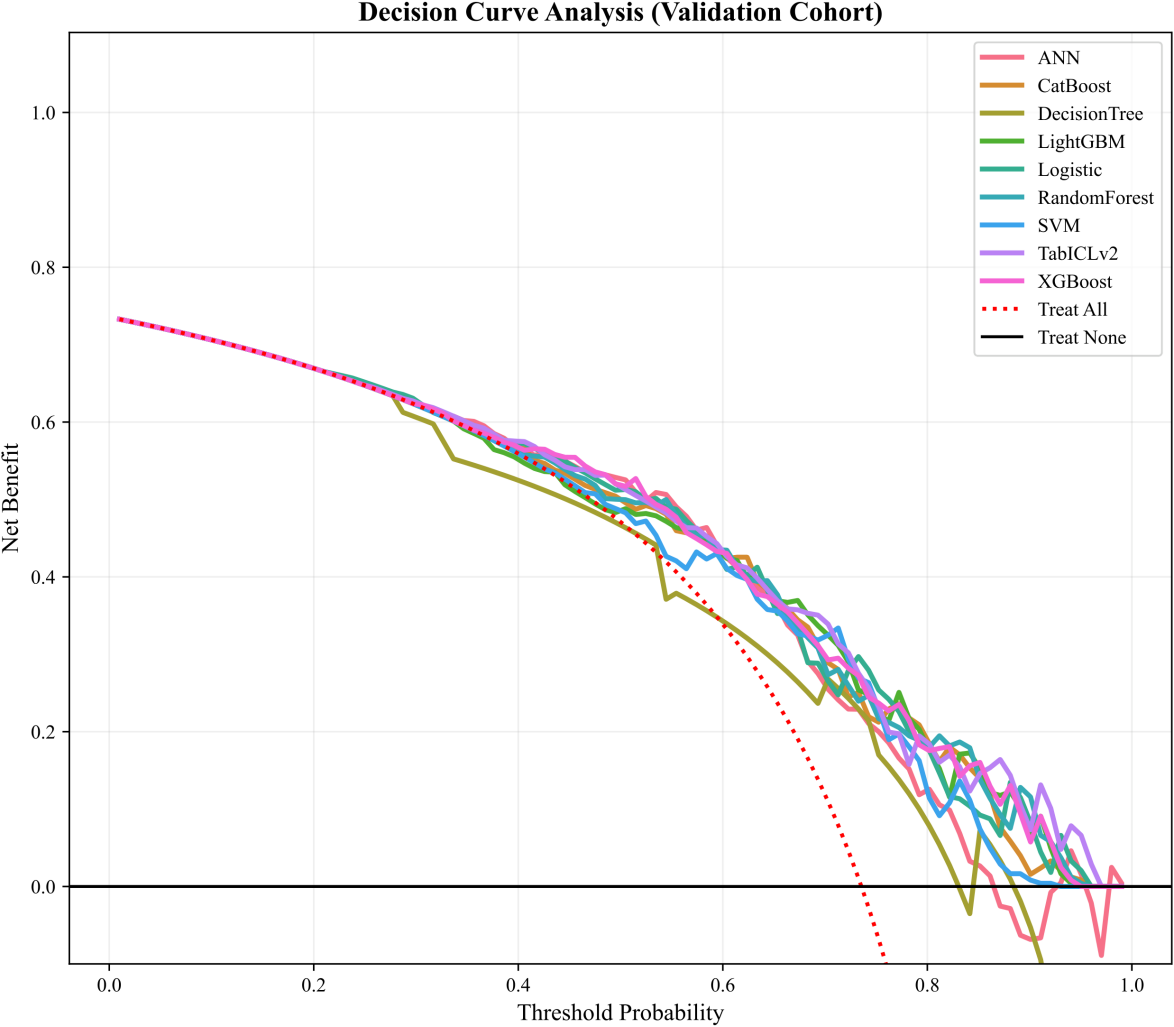
Decision curve analysis (DCA) of the predictive models in the validation cohort. The y-axis represents the clinical net benefit. The graph compares the net benefit of the evaluated machine learning algorithms against the default clinical strategies of “treat-all” (dotted red line) and “treat-none” (solid black horizontal line) across a continuum of threshold probabilities. Abbreviations: DCA, Decision Curve Analysis; TabICLv2, Tabular In-Context Learning Version 2.

Calibration curves (**Supplementary Figure 3**) indicated acceptable probability calibration across the evaluated models. TabICLv2 achieved a Brier score of 0.158, which was slightly lower than those of XGBoost (0.160) and Random Forest (0.163). Decision Curve Analysis (DCA) suggested that TabICLv2 provided a favourable net benefit across clinically relevant threshold probabilities (**Figure 3**). Confusion matrix analysis (**Supplementary Figure 4**) summarised the classification performance of TabICLv2 at the optimal probability threshold.

### 3.5 Sensitivity analyses

To examine whether the composite endpoint was robust to alternative outcome definitions, we repeated the evaluation of TabICLv2 using albumin-only hypoalbuminaemia and total-protein-only hypoproteinaemia as separate endpoints. In the overall cohort, 505 of 812 patients (62.2%) met the albumin-only definition, and 525 of 812 patients (64.7%) met the total-protein-only definition. The two definitions showed substantial overlap: 434 patients met both criteria, whereas 71 met only the albumin criterion and 91 met only the total-protein criterion. For the albumin-only endpoint, TabICLv2 achieved a validation AUC of 0.747 (95% CI: 0.684–0.811) and a Brier score of 0.194. At the optimal threshold of 0.588, the model achieved an accuracy of 0.705, sensitivity of 0.730, specificity of 0.663, and F1 score of 0.755. For the total-protein-only endpoint, the corresponding validation AUC and Brier score were 0.742 (95% CI: 0.675–0.801) and 0.190, respectively. At the optimal threshold of 0.629, the model achieved an accuracy of 0.689, sensitivity of 0.690, specificity of 0.686, and F1 score of 0.741. Compared with the primary composite endpoint, which achieved an AUC of 0.766 (95% CI: 0.699–0.832) and a Brier score of 0.158, discrimination was slightly lower but broadly consistent across the two separate outcome definitions. These findings suggest that the model retained predictive ability for both postoperative hypoalbuminaemia and hypoproteinaemia, while the composite endpoint should be interpreted as acute postoperative protein depletion rather than isolated hypoalbuminaemia alone. The full results are provided in **Supplementary Table 2** and **Supplementary Figure 8**. A complete-case sensitivity analysis was further performed among 572 patients with complete data for all nine selected predictors and the primary outcome, including 404 patients with acute postoperative protein depletion and 168 controls. After applying the same stratified 7:3 training-validation framework, TabICLv2 achieved a validation AUC of 0.736 (95% CI: 0.660–0.809), with an accuracy of 0.640, PPV of 0.940, NPV of 0.448, sensitivity of 0.521, specificity of 0.922, F1 score of 0.670, and Cohen’s kappa of 0.339. Although discrimination was modestly lower than that in the main imputed-data analysis, the result remained broadly comparable, suggesting that the model performance was not solely driven by the imputation strategy (**Supplementary Table 5**).

### 3.6 Model interpretability and clinical feature attribution

**Figure 4:**
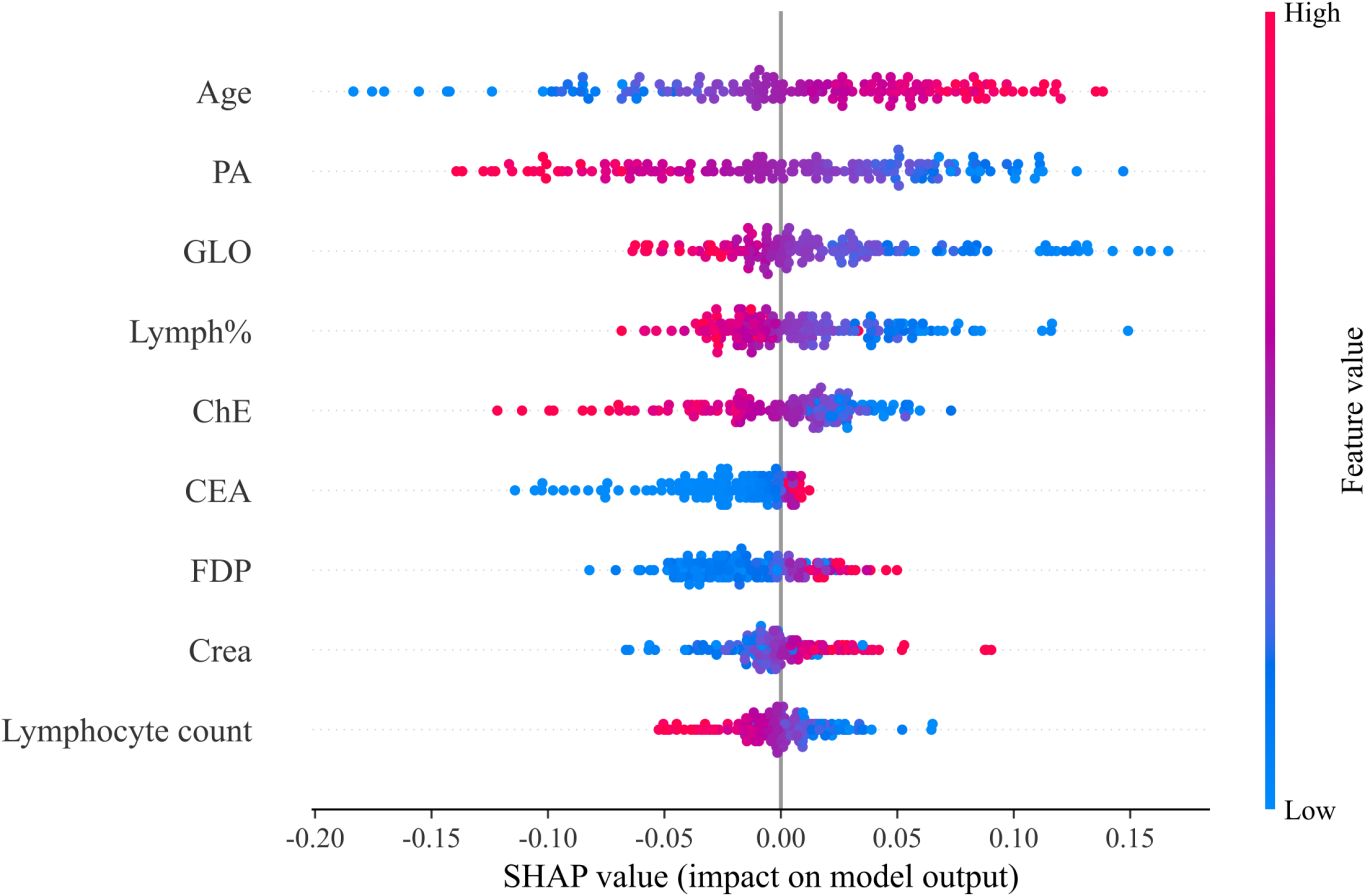
SHAP summary plot (beeswarm plot) of the core predictive features for the TabICLv2 model. Features are ranked on the y-axis in descending order of their global predictive importance. Each dot represents an individual patient. The colour gradient reflects the observed value of the corresponding clinical indicator, with red indicating higher values and blue indicating lower values. The x-axis represents the SHAP value, which quantifies the marginal contribution of each feature to the model output. Positive SHAP values, plotted to the right of the zero reference line, indicate an increased predicted probability of acute postoperative protein depletion, whereas negative SHAP values indicate a decreased predicted probability. Abbreviations: SHAP, SHapley Additive exPlanations; PA, prealbumin; GLO, globulin; Lymph%, lymphocyte percentage; ChE, cholinesterase; CEA, carcinoembryonic antigen; FDP, fibrin degradation products; Crea, creatinine.

A model-agnostic SHAP approach was applied to the TabICLv2 model to elucidate feature contributions. The global summary beeswarm plot (**Figure 4**) identified age, prealbumin (PA), and globulin (GLO) as the top three prognostic factors.

Specifically, lower preoperative PA, GLO, lymphocyte percentage (Lymph%), and cholinesterase (ChE) correlated with positive SHAP values, indicating an increased risk of acute postoperative protein depletion. Conversely, elevated age, fibrin degradation products (FDP), creatinine (Crea), and CEA were associated with increased risk.

SHAP dependence plots with LOWESS smoothing (**Supplementary Figure 5**) revealed non-linear risk trajectories. The risk increased sharply when PA dropped below 200 mg/L, age exceeded 70.8 years, or GLO fell below 30 g/L. FDP demonstrated rapid risk accumulation even with minor elevations.

Exploratory SHAP-based pairwise interaction visualisations further suggested that the risk contribution of nutritional and immune-related predictors varied across clinical contexts. Low PA consistently showed positive SHAP contributions to predicted risk, and this risk pattern appeared more clinically pronounced in the context of advanced age, lower GLO, reduced Lymph%, or lower ChE. Similarly, age-related risk contribution increased with advancing age, while low GLO showed positive SHAP contributions that were more evident in patients with lower lymphocyte counts. These findings support a potential combined contribution of ageing, impaired nutritional reserve, hepatic synthetic dysfunction, and immune-inflammatory vulnerability to acute postoperative protein depletion (**Supplementary Figure 9**).

Localised SHAP waterfall plots illustrated individual risk attribution (**Supplementary Figure 6A, S6B**). From a baseline expected probability of 0.697, an exemplary high-risk case saw their probability increase to 0.892, primarily driven by a decreased Lymph% (7.2%), low PA (139.3 mg/L), and advanced age (73.8 years). In contrast, a low-risk patient was classified at 0.299, supported by adequate nutritional reserves (PA = 311.2 mg/L), normal kidney function (Crea = 50.8 *µ*mol/L), and a younger age (58.8 years).

### 3.7 Development of a web-based prediction platform

#### 3.7.1 Platform construction and deployment

To facilitate clinical translation, a web-based calculator (https://coloncancer-risk-app.streamlit.app/) was developed utilising the optimal TabICLv2 model. The platform integrates the nine core predictors: age, PA, GLO, Lymph%, ChE, CEA, FDP, Crea, and lymphocyte count. The interface computes an individualised absolute risk probability for acute postoperative protein depletion and generates real-time SHAP visualisations (waterfall, force, decision, and bar plots) to delineate the directional impact and magnitude of each variable for the specific patient.

#### 3.7.2 Clinical application example

A representative preoperative scenario illustrates the platform’s standard output (**Supplementary Figure 7**). The baseline input parameters for this case were: age = 73.3 years, PA = 139 mg/L, Lymph% = 7.6%, ChE = 4000 U/L, Crea = 20 *µ*mol/L, GLO = 25 g/L, lymphocyte count = 0.62 × 10^9^/L, FDP = 22.9 mg/L, and CEA = 311.5 ng/mL. Based on these inputs, the application computed a predicted probability of 93.04% for acute postoperative protein depletion. The corresponding personalised SHAP waterfall plot indicated that the patient’s decreased Lymph% (7.6%), low PA (139 mg/L), and reduced ChE (4000 U/L) were the primary drivers underlying this elevated risk. Additionally, the localised SHAP plot demonstrated that the specific Crea and FDP levels yielded negative SHAP values, marginally mitigating the overall risk estimate.

## 4 Discussion

In this retrospective cohort study, we developed and validated a risk prediction model for acute postoperative protein depletion in 812 patients undergoing colon cancer surgery. This study represents an exploratory application of the TabICLv2 tabular foundation model to surgery-related metabolic risk prediction. TabICLv2 achieved the numerically highest validation AUC point estimate and showed comparable discrimination to the strongest conventional machine learning classifiers, although paired DeLong tests did not demonstrate statistically significant AUC differences compared with XGBoost or Random Forest. Complementary NRI and IDI analyses suggested improved event reclassification across comparator models and significant total NRI/IDI improvement over Random Forest, whereas incremental improvement over Logistic Regression and XGBoost was mainly confined to event reclassification. Sensitivity analyses showed broadly consistent discrimination when albumin-only and total-protein-only endpoints were evaluated separately, supporting the robustness of the model while clarifying that the composite endpoint should be interpreted as acute postoperative protein depletion rather than isolated hypoalbuminaemia alone. The accompanying web-based application may support early identification of high-risk patients and assist perioperative nutritional risk assessment, but prospective external validation is required before routine clinical implementation.

Despite the widespread implementation of Enhanced Recovery After Surgery (ERAS) protocols (13), the incidence of postoperative hypoalbuminaemia following colon resection remains remarkably high (14). Acute postoperative albumin depletion reflects the magnitude of surgical stress and is intrinsically linked to severe complications, such as anastomotic leakage, surgical site infections, and prolonged hospitalisation (15–17). In our study, SHAP analysis identified age, prealbumin (PA), and globulin (GLO) as the dominant predictive drivers, underscoring that acute postoperative protein depletion is predominantly governed by the interplay among age-related physiological decline, baseline nutritional reserves, and the immuno-inflammatory milieu.

Preoperative nutritional optimisation is a cornerstone of perioperative management for colon cancer surgery (18, 19). Our findings reaffirm PA as a critical prognosticator. While serum albumin has a half-life of approximately 20 days, PA circulates for only 2 to 3 days (20). This metabolic distinction allows PA to capture acute fluctuations in protein synthesis and catabolism much more sensitively than albumin (21, 22). Extant literature supports that depressed preoperative PA independently predicts severe post-colectomy complications, often outperforming both body mass index and baseline albumin (23). Our non-linear threshold analysis elaborates on this dynamic, revealing a sharp escalation in risk when PA concentrations fall below 200 mg/L. Consequently, patients exhibiting “hidden malnutrition”—defined by suppressed PA in the presence of normal baseline albumin—warrant proactive, targeted nutritional prehabilitation.

Notably, chronological age emerged as the paramount predictor within the TabICLv2 framework. The SHAP dependence analysis revealed a distinct risk inflection point at approximately 70.8 years of age, illustrating the threshold at which reduced physiological reserve may become more apparent in older patients. Advancing age is inextricably linked with sarcopenia, altered basal protein turnover, and an attenuated homeostatic response to surgical stress, rendering older patients highly susceptible to precipitous postoperative protein loss.

Equally critical is the preoperative immuno-inflammatory profile, primarily represented in our model by GLO and lymphocyte indices. As a surrogate marker for systemic humoral immunity, depressed baseline GLO (particularly below the identified 30 g/L threshold) signifies an impaired immunological reserve. When compounded by the surgical stress response and subsequent systemic inflammatory response syndrome (SIRS), capillary permeability increases drastically. This drives the rapid extravasation of albumin from the intravascular compartment into the interstitium, recognised as an elevated transcapillary escape rate (24–26). Furthermore, tumour-induced systemic consumption often leads to the concurrent depletion of GLO and lymphocytes, reflecting a state of severe immune exhaustion and hypercatabolism that actively downregulates hepatic albumin synthesis (27, 28). This interpretation is also consistent with recent oncology studies emphasising that tumour progression and treatment vulnerability are shaped by complex interactions among tumour cells, immune-inflammatory signalling, neural regulation, post-translational modification, and the tumour microenvironment (35, 37, 38). Although these studies focussed on different cancer types or mechanistic contexts, they support the broader concept that perioperative metabolic risk cannot be fully understood from isolated laboratory indicators alone.

Integrating hepatic synthesis parameters (ChE) and coagulation markers (FDP) into the predictive framework yielded additional mechanistic insights. Patients with gastrointestinal malignancies frequently exhibit a baseline hypercoagulable state (29). Elevated FDP indicates active hyperfibrinolysis and correlates strongly with an increased tumour burden (30). Recent evidence highlights an extensive “crosstalk” between the coagulation cascade and inflammatory pathways; the activation of coagulation factors stimulates the release of pro-inflammatory cytokines, which in turn indirectly suppress hepatic albumin transcription (31). These multifaceted findings emphasise that perioperative nutritional indices cannot be evaluated in isolation. Advanced, transparent prediction models are essential to comprehensively assess and simultaneously manage the compounding metabolic detriments of advanced age, immunosuppression, and coagulopathy.

The exploratory pairwise SHAP visualisations further support this integrative interpretation. Rather than indicating that acute postoperative protein depletion is driven by a single biomarker, the observed patterns suggest that reduced PA, low GLO, lymphocyte depletion, impaired ChE, and advanced age may jointly shape postoperative protein-depletion risk. This finding is clinically plausible because baseline nutritional reserve, hepatic synthetic capacity, immune-inflammatory status, and age-related physiological vulnerability may amplify one another during the postoperative stress response.

From a clinical perspective, the validation AUC of 0.766 and Cohen’s Kappa of 0.393 indicate moderate rather than excellent predictive performance. Although no universally accepted minimum AUC threshold exists for perioperative nutritional risk prediction, a clinically useful screening model should provide sufficient discrimination, acceptable calibration, and demonstrable clinical net benefit to support targeted monitoring or intervention. Therefore, the present model should be regarded as a risk-stratification and decision-support tool rather than a standalone diagnostic system. Its primary role is to assist early identification of patients who may benefit from closer postoperative biochemical monitoring, early nutritional assessment, and perioperative optimisation, rather than to replace clinical judgement. The web-based calculator is intended for adult patients with primary colon cancer undergoing elective radical resection when all nine required preoperative predictors are available. It should not be used as a standalone decision-making tool, and its applicability to emergency surgery, rectal cancer, metastatic disease, severe baseline hepatic or renal dysfunction, or institutions with substantially different perioperative practices requires further validation. In clinical practice, high predicted risk may prompt closer postoperative biochemical monitoring, early nutritional assessment, and consideration of perioperative nutritional optimisation, but specific intervention thresholds should be evaluated prospectively. From a supportive-care perspective, the clinical translation of risk prediction models should also be interpreted within the broader context of individualised cancer management, where translational systems and nutrition-related strategies have been increasingly explored to support personalised treatment and supportive care (33, 39).

Several limitations should be acknowledged. First, this was a single-centre retrospective study based on consecutive eligible patients from one institution. Therefore, the observed predictor distributions, laboratory measurement protocols, perioperative nutritional practices, surgical pathways, and postoperative monitoring strategies may not fully represent those of other hospitals or healthcare systems. As a result, the generalisability of the present model is limited, and the findings may not be directly applicable to external centres without further validation. In addition, because no formal *a priori* sample size calculation was performed and the initial candidate feature pool was relatively large, residual overfitting risk cannot be completely excluded despite the sufficient number of outcome events relative to the final predictors.

Second, training-validation AUC difference analysis showed statistically significant decreases for several flexible models, including Decision Tree, Random Forest, LightGBM, CatBoost, and TabICLv2, suggesting apparent optimism and potential overfitting. This may be related to the moderate sample size, high initial candidate-feature dimensionality, model flexibility, data-driven recursive feature elimination, and the use of development-cohort predictions when estimating training performance. Accordingly, validation-cohort performance was emphasised as the primary basis for model comparison. Although feature selection was restricted to the training cohort and five-fold cross-validation was used during model development, temporal validation, external validation, and ideally prospective multicentre validation are still required to assess generalisability across institutions and time periods. Third, the model demonstrated moderate rather than excellent predictive performance, with a validation AUC of 0.766 and Cohen’s Kappa of 0.393. Therefore, it should be considered an adjunctive risk-stratification tool rather than a definitive diagnostic or treatment-decision system. Its current applicable population is restricted to adult patients with primary colon cancer undergoing elective radical resection and with all nine required preoperative predictors available. The applicability of this model to emergency surgery, rectal cancer, metastatic disease, patients with severe baseline hepatic or renal dysfunction, and institutions with substantially different perioperative pathways remains uncertain. Fourth, the SHAP-based pairwise interaction analysis was exploratory and visual in nature. Because TabICLv2 is not a tree-ensemble model, exact TreeSHAP interaction values were not calculated. Therefore, the observed pairwise patterns should be interpreted as hypothesis-generating rather than confirmatory evidence of statistical interaction, and they require validation in larger external cohorts.

## 5 Conclusion

In conclusion, this study developed and internally validated a TabICLv2-based prediction model for acute postoperative protein depletion in patients undergoing colon cancer surgery. Using nine preoperative variables, including age, prealbumin, and globulin, the model achieved moderate discrimination and acceptable calibration in the validation cohort, with performance comparable to that of the strongest conventional machine learning models. NRI and IDI analyses suggested improved event-risk reclassification and modest incremental risk-stratification benefit, particularly compared with Random Forest, although overall improvement over Logistic Regression and XGBoost remained limited. SHAP-based interpretation provided clinically plausible risk attribution, and the accompanying web application may facilitate adjunctive individualised perioperative risk assessment. Prospective, multicentre validation remains necessary to confirm generalisability and clinical applicability.

## Data Availability

The raw data supporting the conclusions of this article will be made available by the authors, without undue reservation.
