# Supplementary material for: Prediction of acute postoperative protein depletion risk in colon cancer using an in-context learning foundation model: a retrospective cohort study
Source: Front Oncol. 2026 Jul 8;16:1857275. doi: 10.3389/fonc.2026.1857275 (PMC13388145; doi:10.3389/fonc.2026.1857275)
Supplement: Supplementary file 1 [file DataSheet1.pdf]

Supplementary Materials

Supplementary Table S1. Hyperparameter search spaces and optimal configurations for the evaluated machine learning models.

Machine Learning Model	Optimisation Method	Search Space	Optimal Hyperparameters
**Random Forest**	Optuna	n_estimators: 50–500, step 50; max_depth: 3–20; min_samples_split: 2–20; min_samples_leaf: 1–20; max_features: sqrt, log2, or None	n_estimators: 250; max_depth: 4; min_samples_split: 10; min_samples_leaf: 8; max_features: log2
**XGBoost**	Optuna	n_estimators: 50–600, step 50; max_depth: 2–7; min_child_weight: 1–10; learning_rate: 0.001–0.300, log scale; subsample: 0.60–1.00; colsample_bytree: 0.60–1.00; gamma: 0.0001–5.0000, log scale; reg_alpha: 0.0001–10.0000, log scale; reg_lambda: 0.0001–10.0000, log scale	n_estimators: 150; max_depth: 2; min_child_weight: 6; learning_rate: 0.02700; subsample: 0.85688; colsample_bytree: 0.99691; gamma: 4.46647; reg_alpha: 0.00103; reg_lambda: 0.00076
**LightGBM**	Optuna	n_estimators: 50–500, step 50; learning_rate: 0.010–0.300, log scale; num_leaves: 20–150; max_depth: 3–12; subsample: 0.50–1.00; colsample_bytree: 0.50–1.00; reg_alpha: 0–5; reg_lambda: 0–5	n_estimators: 100; learning_rate: 0.04616; num_leaves: 62; max_depth: 11; subsample: 0.80762; colsample_bytree: 0.55576; reg_alpha: 4.74444; reg_lambda: 2.53388
**SVM**	Optuna	C: 0.001–100.000, log scale; gamma: scale or auto; kernel: rbf or linear	kernel: linear; C: 10.20205; gamma: scale
**Decision Tree**	Optuna	max_depth: 3–20; min_samples_split: 2–50; min_samples_leaf: 1–50; criterion: gini or entropy	max_depth: 10; min_samples_split: 42; min_samples_leaf: 26; criterion: gini
**ANN**	Optuna	hidden_layer_sizes: (50,), (100,), (50, 50), or (100, 50); activation: relu or tanh; solver: adam or sgd; alpha: 0.00001–0.10000, log scale; learning_rate_init: 0.00010–0.10000, log scale; max_iter: 500	hidden_layer_sizes: (50,); activation: relu; solver: sgd; alpha: 0.00036; learning_rate_init: 0.00285

Continued on next page

Machine Learning Model	Optimisation Method	Search Space	Optimal Hyperparameters
**CatBoost**	Optuna	iterations: 100–1000, step 100; depth: 3–10; learning_rate: 0.001–0.300, log scale; l2_leaf_reg: 0.010–10.000, log scale; border_count: 32–255	iterations: 900; depth: 5; learning_rate: 0.00205; l2_leaf_reg: 0.05178; border_count: 77
**TabICLv2**	Not applicable	Not applicable; pre-trained tabular foundation model used without dataset-specific hyperparameter search	Pre-trained tabular in-context inference architecture deployed out-of-the-box; no dataset-specific hyperparameter tuning required

Note: Hyperparameter optimisation for conventional machine learning models was performed using Optuna with five-fold cross-validation in the training cohort, using AUC as the optimisation objective. The search spaces shown represent the candidate ranges or categorical options evaluated during optimisation. Random Forest, ANN, CatBoost, and SVM were optimised using 30, 30, 30, and 20 Optuna trials, respectively, whereas XGBoost, LightGBM, and Decision Tree were optimised using 50 trials. ANN and SVM were trained after standardisation using StandardScaler fitted on the training cohort and then applied to the validation cohort. TabICLv2 was not subjected to dataset-specific hyperparameter optimisation because it was applied as a pre-trained tabular foundation model. ANN, artificial neural network; SVM, support vector machine; XGBoost, Extreme Gradient Boosting; LightGBM, Light Gradient Boosting Machine; CatBoost, Categorical Boosting; TabICLv2, Tabular In-Context Learning Version 2.

Supplementary Table S2. Sensitivity analysis of TabICLv2 performance using alternative outcome definitions in the validation cohort.

Endpoint	Optimal threshold	AUC (95% CI)	Metrics at optimal threshold	Brier score
**Composite endpoint**	0.689	0.766 (0.699–0.832)	Accuracy: 0.736; PPV: 0.870; sensitivity: 0.753; specificity: 0.688; F1 score: 0.807; Kappa: 0.393; NPV: 0.500	0.158
**Albumin-only endpoint**	0.588	0.747 (0.684–0.811)	Accuracy: 0.705; PPV: 0.782; sensitivity: 0.730; specificity: 0.663; F1 score: 0.755; Kappa: 0.385; NPV: 0.598	0.194
**Total-protein-only endpoint**	0.629	0.742 (0.675–0.801)	Accuracy: 0.689; PPV: 0.801; sensitivity: 0.690; specificity: 0.686; F1 score: 0.741; Kappa: 0.355; NPV: 0.546	0.190

Note: The composite endpoint was defined as either serum albumin < 35 g/L or serum total protein < 60 g/L during the first postoperative week. The albumin-only endpoint was defined as serum albumin < 35 g/L, and the total-protein-only endpoint was defined as serum total protein < 60 g/L. Optimal thresholds were determined using the Youden Index in the validation cohort. **Abbreviations:** AUC, area under the receiver operating characteristic curve; CI, confidence interval; PPV, positive predictive value; NPV, negative predictive value.

Supplementary Table S3. Pairwise DeLong tests comparing the validation AUC of TabICLv2 with the strongest conventional machine learning models.

Comparison	AUC 1	AUC 2	AUC difference	95% CI for difference	*P* value
TabICLv2 vs XGBoost	0.766	0.763	0.003	-0.030 to 0.037	0.854
TabICLv2 vs Random Forest	0.766	0.760	0.006	-0.024 to 0.035	0.712

Note: AUC differences were calculated as TabICLv2 minus the comparator model. Two-sided *P* values were obtained using paired DeLong tests because predictions were compared within the same validation cohort. Abbreviations: AUC, area under the receiver operating characteristic curve; CI, confidence interval.

Supplementary Table S4. Variables excluded because of missingness greater than 30%.

Variable	Missing rate	Handling strategy
FFA	62.7%	Excluded from model development and main baseline table
LPa	42.7%	Excluded from model development and main baseline table
TG	42.6%	Excluded from model development and main baseline table
apoB	42.6%	Excluded from model development and main baseline table
apoA-I	42.5%	Excluded from model development and main baseline table
HDL-C	42.2%	Excluded from model development and main baseline table
TC	42.1%	Excluded from model development and main baseline table
LDL-C	42.0%	Excluded from model development and main baseline table
CRP	39.2%	Excluded from model development and main baseline table
RBP	33.3%	Excluded from model development and main baseline table
AFU	31.7%	Excluded from model development and main baseline table

Note: Variables with missingness greater than 30% were excluded according to the predefined missing-data rule. Abbreviations: FFA, free fatty acids; LPa, lipoprotein(a); TG, triglyceride; apoB, apolipoprotein B; apoA-I, apolipoprotein A-I; HDL-C, high-density lipoprotein cholesterol; TC, total cholesterol; LDL-C, low-density lipoprotein cholesterol; CRP, C-reactive protein; RBP, retinol-binding protein; AFU, alpha-L-fucosidase.

Supplementary Table S5. Complete-case sensitivity analysis of the TabICLv2 model.

Analysis	AUC (95% CI)	Threshold	Accuracy	PPV	NPV	Sensitivity	Specificity
Main imputed analysis	0.766 (0.699–0.832)	0.689	0.736	0.870	0.500	0.753	0.688
Complete-case analysis	0.736 (0.660–0.809)	0.828	0.640	0.940	0.448	0.521	0.922

Note: The complete-case analysis was performed among patients with complete data for the final nine predictors and the primary outcome, without missing-value imputation. The main imputed analysis was based on the MICE-imputed dataset. Abbreviations: AUC, area under the receiver operating characteristic curve; CI, confidence interval; MICE, Multiple Imputation by Chained Equations; PPV, positive predictive value; NPV, negative predictive value.

Supplementary Table S6. Univariable logistic regression analysis of baseline variables associated with acute postoperative protein depletion.

Variable	Term	N	Events	OR (95% CI)	*P* value
PA	Per 1-unit increase	812	596	0.990 (0.988–0.993)	< 0.001
ChE	Per 1-unit increase	812	596	1.000 (1.000–1.000)	< 0.001
Ca	Per 1-unit increase	812	596	0.011 (0.003–0.040)	< 0.001
ALB	Per 1-unit increase	812	596	0.883 (0.851–0.915)	< 0.001
Age	Per 12-month increase	812	596	1.045 (1.031–1.059)	< 0.001
TP	Per 1-unit increase	812	596	0.941 (0.921–0.961)	< 0.001
Lymphocyte count	Per 1-unit increase	812	596	0.491 (0.372–0.647)	< 0.001
FDP	Per 1-unit increase	812	596	1.191 (1.107–1.282)	< 0.001
ATIII:A	Per 1-unit increase	812	596	0.975 (0.965–0.985)	< 0.001
eGFR	Per 1-unit increase	812	596	0.975 (0.965–0.986)	< 0.001
D-Dimer	Per 1-unit increase	812	596	1.001 (1.001–1.002)	< 0.001
Lymph%	Per 1-unit increase	812	596	0.963 (0.947–0.980)	< 0.001
AST/ALT	Per 1-unit increase	812	596	2.094 (1.481–2.960)	< 0.001
Fbg	Per 1-unit increase	812	596	1.596 (1.272–2.003)	< 0.001
PT	Per 1-unit increase	812	596	1.364 (1.147–1.623)	< 0.001
TBA	Per 1-unit increase	812	596	1.113 (1.047–1.182)	< 0.001
Neut%	Per 1-unit increase	812	596	1.025 (1.010–1.041)	0.001
Na^{+}	Per 1-unit increase	812	596	0.893 (0.834–0.956)	0.001
Hb	Per 1-unit increase	812	596	0.990 (0.985–0.996)	0.001
CA125	Per 1-unit increase	812	596	1.017 (1.006–1.028)	0.002
Hct	Per 1-unit increase	812	596	0.964 (0.942–0.987)	0.002
RBC	Per 1-unit increase	812	596	0.685 (0.534–0.877)	0.003
RDW-CV	Per 1-unit increase	812	596	1.117 (1.038–1.202)	0.003
UA	Per 1-unit increase	812	596	0.997 (0.996–0.999)	0.003
PT-INR	Per 1-unit increase	812	596	17.254 (2.435–122.249)	0.004
Mg^{2+}	Per 1-unit increase	812	596	0.033 (0.003–0.362)	0.005
Baso%	Per 1-unit increase	812	596	0.440 (0.244–0.793)	0.006
MCHC	Per 1-unit increase	812	596	0.989 (0.981–0.997)	0.007
ALP	Per 1-unit increase	812	596	1.009 (1.002–1.015)	0.008
ALT	Per 1-unit increase	812	596	0.982 (0.967–0.997)	0.016
Baso	Per 1-unit increase	812	596	0.000 (0.000–0.157)	0.018
AST	Per 1-unit increase	812	596	1.017 (1.002–1.033)	0.023

Continued on next page

**Variable**	**Term**	**N**	**Events**	**OR (95% CI)**	***P* value**
Mono%	Per 1-unit increase	812	596	1.085 (1.010–1.166)	0.026
Mono	Per 1-unit increase	812	596	2.409 (1.085–5.350)	0.031
Cys-C	Per 1-unit increase	812	596	2.034 (1.039–3.982)	0.038
MCH	Per 1-unit increase	812	596	0.959 (0.922–0.998)	0.039
CEA	Per 1-unit increase	812	596	1.007 (1.000–1.014)	0.039
GLO	Per 1-unit increase	812	596	0.968 (0.938–0.999)	0.042
P	Per 1-unit increase	812	596	0.431 (0.186–0.999)	0.050
Glu	Per 1-unit increase	812	596	1.113 (1.000–1.238)	0.050
ALB/GLO	Per 1-unit increase	812	596	0.573 (0.316–1.039)	0.067
Cl^-	Per 1-unit increase	812	596	0.955 (0.908–1.005)	0.075
Osm	Per 1-unit increase	812	596	0.973 (0.941–1.006)	0.107
CO_2CP	Per 1-unit increase	812	596	0.952 (0.895–1.013)	0.120
LDH	Per 1-unit increase	812	596	1.002 (1.000–1.004)	0.121
AFP	Per 1-unit increase	812	596	1.073 (0.976–1.179)	0.144
Pulse	Per 1-unit increase	812	596	1.011 (0.996–1.026)	0.149
CA19-9	Per 1-unit increase	812	596	1.001 (0.999–1.003)	0.161
MCV	Per 1-unit increase	812	596	0.987 (0.969–1.005)	0.169
APTT	Per 1-unit increase	812	596	0.966 (0.918–1.016)	0.182
Heart rate	Per 1-unit increase	812	596	1.010 (0.995–1.025)	0.191
Diabetes	Yes vs No	812	596	1.367 (0.845–2.213)	0.203
Urea	Per 1-unit increase	812	596	1.058 (0.968–1.157)	0.211
Crea	Per 1-unit increase	812	596	1.005 (0.997–1.014)	0.213
GGT	Per 1-unit increase	812	596	0.997 (0.991–1.003)	0.282
Neut	Per 1-unit increase	812	596	1.040 (0.967–1.119)	0.293
TT	Per 1-unit increase	812	596	0.966 (0.902–1.034)	0.316
ADA	Per 1-unit increase	812	596	1.025 (0.977–1.075)	0.320
Hypertension	Yes vs No	812	596	1.171 (0.853–1.607)	0.328
DBIL	Per 1-unit increase	812	596	1.043 (0.958–1.136)	0.328
PDW	Per 1-unit increase	812	596	0.973 (0.911–1.038)	0.405
Diastolic blood pressure	Per 1-unit increase	812	596	0.994 (0.978–1.010)	0.437
CHD	Yes vs No	812	596	1.465 (0.543–3.954)	0.451
PLT	Per 1-unit increase	812	596	1.001 (0.999–1.002)	0.504
MPV	Per 1-unit increase	812	596	0.952 (0.821–1.104)	0.519
Platelet volume	Per 1-unit increase	812	596	1.835 (0.285–11.812)	0.523

Continued on next page

Supplementary Table S6 – Continued from previous page

Variable	Term	N	Events	OR (95% CI)	P value
AG	Per 1-unit increase	812	596	1.020 (0.952–1.092)	0.577
Gender	Female vs Male	812	596	0.922 (0.673–1.263)	0.612
Systolic blood pressure	Per 1-unit increase	812	596	1.002 (0.993–1.012)	0.619
Urea/Crea	Per 1-unit increase	812	596	3.819 (0.007–2207.641)	0.680
K^+	Per 1-unit increase	812	596	0.944 (0.645–1.380)	0.765
Temperature	Per 1-unit increase	812	596	0.942 (0.582–1.525)	0.809
TBIL	Per 1-unit increase	812	596	0.998 (0.974–1.021)	0.839
Eos%	Per 1-unit increase	812	596	1.010 (0.915–1.116)	0.843
WBC	Per 1-unit increase	812	596	0.996 (0.934–1.063)	0.914
Eos	Per 1-unit increase	812	596	0.996 (0.231–4.305)	0.996

Note: OR, odds ratio; CI, confidence interval. Each baseline variable listed in Table 1 was entered separately into a univariable logistic regression model. For continuous variables, ORs are presented per 1-unit increase unless otherwise specified. Age was analysed per 12-month increase. Categorical variables were analysed using the absence category or male sex as the reference group, as appropriate. Univariable analyses were exploratory and were not used for machine-learning feature selection.

Supplementary Table S7. Continuous net reclassification improvement and integrated discrimination improvement comparing TabICLv2 with conventional models in the validation cohort.

Comparator	NRI event	NRI non-event	Total NRI	IDI
Logistic Regression	0.315 (0.169–0.458); $P < 0.001$	-0.156 (-0.393–0.088); $P = 0.235$	0.158 (-0.109–0.433); $P = 0.259$	0.006 (-0.014–0.024); $P = 0.510$
Random Forest	0.337 (0.212–0.474); $P < 0.001$	0.156 (-0.077–0.391); $P = 0.229$	0.493 (0.228–0.782); $P < 0.001$	0.035 (0.017–0.053); $P < 0.001$
XGBoost	0.303 (0.170–0.442); $P < 0.001$	-0.125 (-0.356–0.123); $P = 0.342$	0.178 (-0.097–0.462); $P = 0.223$	0.012 (-0.006–0.032); $P = 0.220$

Note: NRI, net reclassification improvement; IDI, integrated discrimination improvement. Continuous/category-free NRI and IDI were calculated using predicted probabilities in the validation cohort. Positive values indicate improvement for TabICLv2 over the comparator model. Confidence intervals and two-sided P values were estimated using bootstrap resampling with 2000 iterations. Logistic Regression, Random Forest, and XGBoost were selected as representative conventional comparator models.

Supplementary Table S8. Apparent training-validation AUC differences of the evaluated models.

Model	Apparent training AUC	Validation AUC	AUC difference (95% CI)	P value	Interpretation
Logistic Regression	0.744	0.755	-0.011 (-0.086–0.076)	0.817	No significant AUC decrease
SVM	0.719	0.745	-0.026 (-0.109–0.062)	0.555	No significant AUC decrease
Decision Tree	0.790	0.681	0.109 (0.064–0.230)	< 0.001	Significant AUC decrease
Random Forest	0.850	0.760	0.090 (0.045–0.198)	0.001	Significant AUC decrease
ANN	0.809	0.734	0.075 (-0.012–0.158)	0.090	No significant AUC decrease
LightGBM	0.861	0.754	0.107 (0.058–0.204)	0.001	Significant AUC decrease
CatBoost	0.917	0.755	0.162 (0.121–0.265)	< 0.001	Significant AUC decrease
XGBoost	0.820	0.763	0.057 (-0.004–0.149)	0.070	No significant AUC decrease
TabICLv2	0.860	0.766	0.094 (0.049–0.195)	< 0.001	Significant AUC decrease

Note: Apparent training AUC was obtained by applying the final fitted model to the training cohort, whereas validation AUC was calculated in the independent validation cohort. AUC difference was calculated as apparent training-cohort AUC minus validation-cohort AUC. Positive values indicate higher apparent discrimination in the training cohort than in the validation cohort and may reflect apparent optimism. Confidence intervals and two-sided P values for the AUC difference were estimated using bootstrap resampling with 2000 iterations, with separate resampling of the training and validation cohorts. AUC, area under the receiver operating characteristic curve; CI, confidence interval; ANN, artificial neural network; SVM, support vector machine; LightGBM, Light Gradient Boosting Machine; XGBoost, Extreme Gradient Boosting; TabICLv2, Tabular In-Context Learning Version 2.

Supplementary Figures

Supplementary Figure S1. Pearson correlation matrix of continuous baseline variables. The colour gradient reflects the Pearson correlation coefficient (r), with red indicating positive correlations and blue indicating negative correlations. Highly correlated feature pairs ($|r| \geq 0.70$) were identified for subsequent selective pruning.

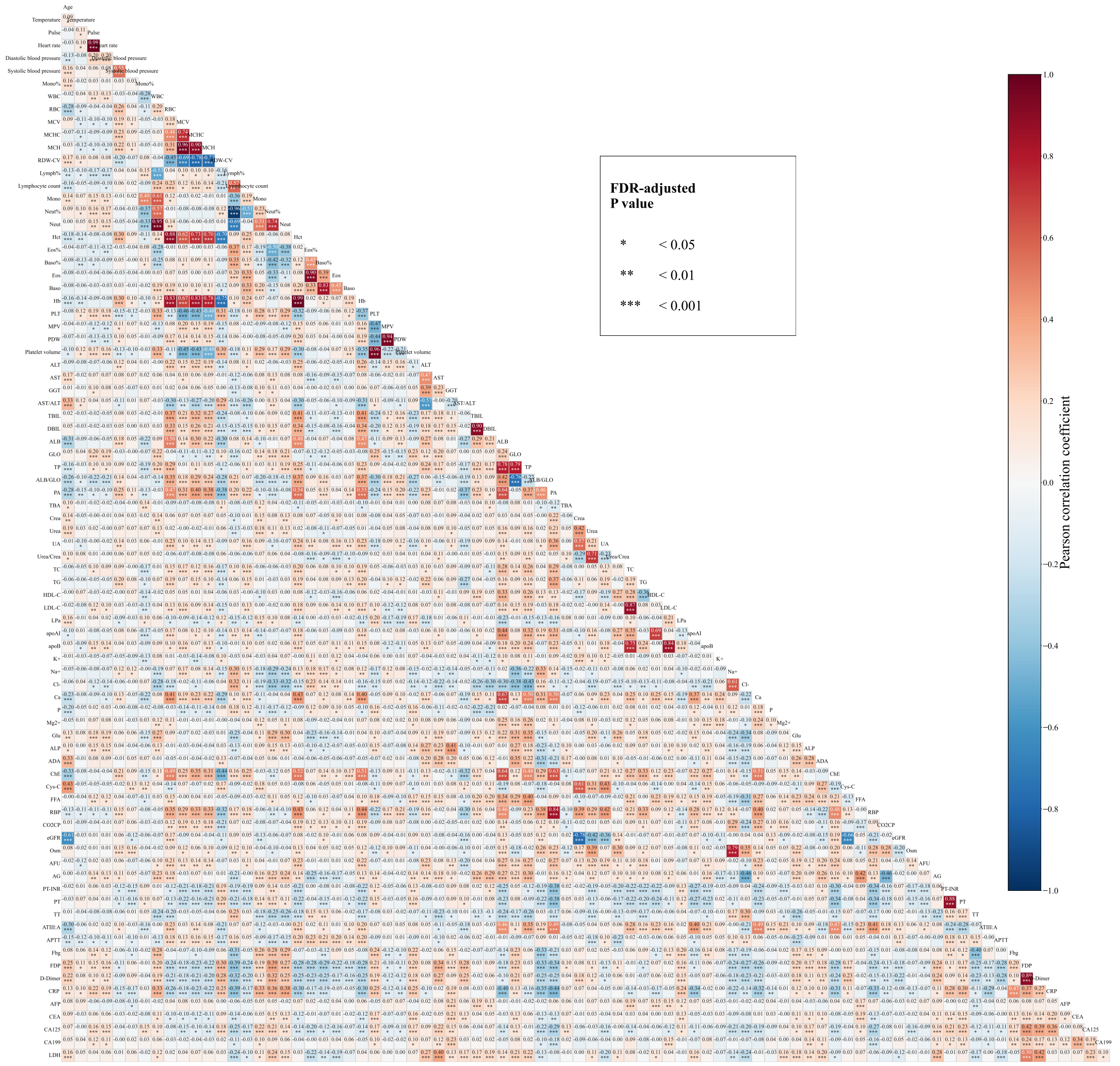
FDR-adjusted
P value
* < 0.05
** < 0.01
*** < 0.001
Pearson correlation coefficient

Supplementary Figure S2. Feature selection using recursive feature elimination (RFE). The curve illustrates the cross-validation accuracy as a function of the number of retained features. The optimal, non-redundant subset of nine core predictors was identified at the point of accuracy stabilisation.

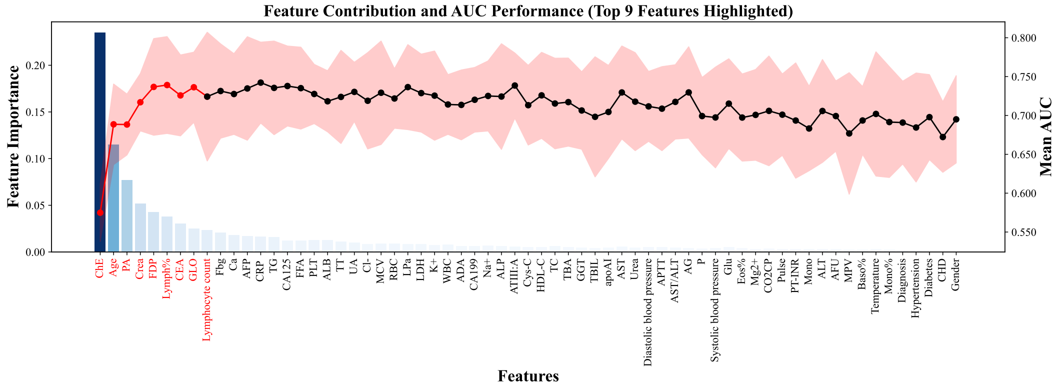

Supplementary Figure S3. Calibration curves of the evaluated predictive models in the validation cohort. The x-axis represents the predicted probability of acute postoperative protein depletion, and the y-axis indicates the actual observed frequency. The diagonal dashed line represents the ideal reference for perfect calibration.

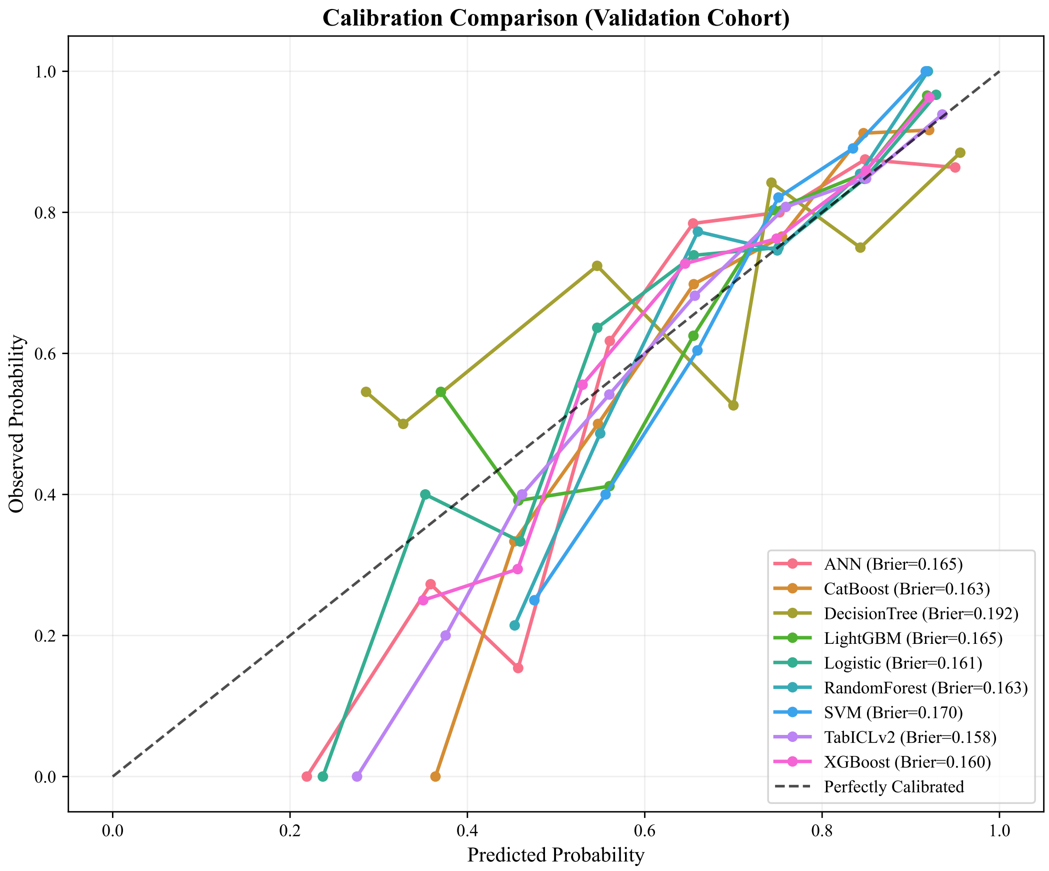

Abbreviations: ANN, Artificial Neural Network; SVM, Support Vector Machine; LightGBM, Light Gradient Boosting Machine; XGBoost, Extreme Gradient Boosting; TabICLv2, Tabular In-Context Learning Version 2.

Supplementary Figure S4. Confusion matrix for the TabICLv2 foundation model in the validation cohort. The matrix displays the absolute patient counts for true negatives (n=44), false positives (n=20), false negatives (n=44), and true positives (n=134), based on the optimal classification threshold determined by the Youden Index.

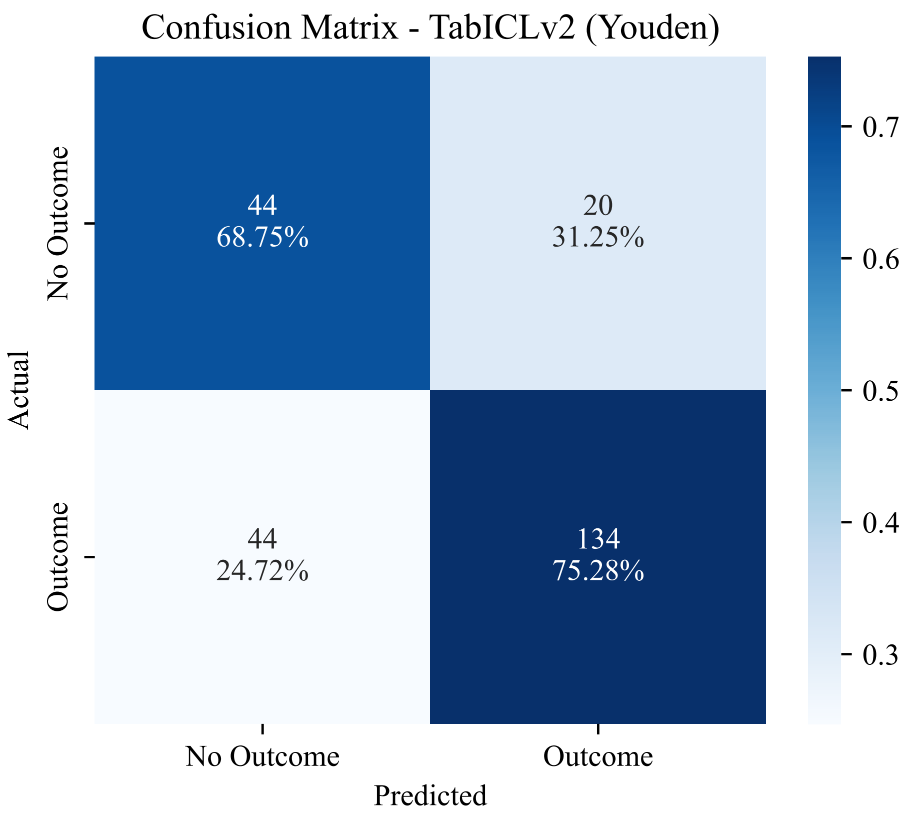

Supplementary Figure S5. SHAP dependence plots for the core predictive features of the TabICLv2 model. Each scatter plot shows the relationship between a specific clinical feature value on the x-axis and its corresponding SHAP value on the y-axis. LOWESS (locally weighted scatterplot smoothing) curves are overlaid to show non-linear trends. A SHAP value > 0 indicates an increased predicted probability of acute postoperative protein depletion, whereas a SHAP value < 0 indicates a decreased predicted probability. The subpanels, from left to right and top to bottom, correspond to the following features: ChE, Age, PA, Crea, FDP, Lymph%, CEA, GLO, and lymphocyte count.

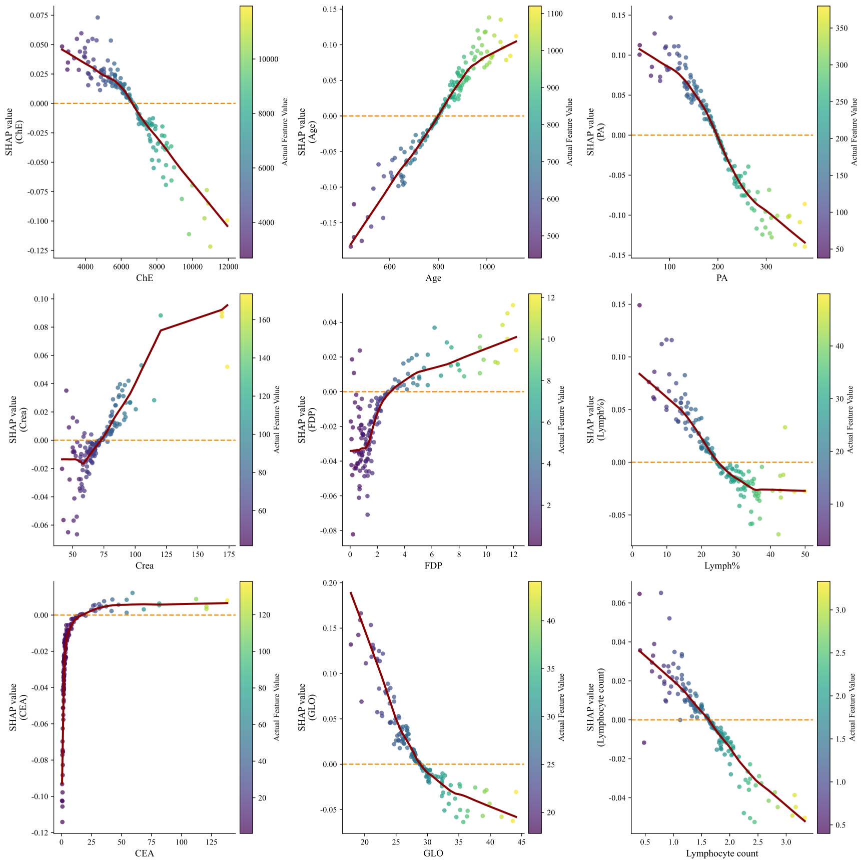

Abbreviations: CEA, carcinoembryonic antigen; ChE, cholinesterase; Crea, creatinine; FDP, fibrin degradation products; GLO, globulin; LOWESS, locally weighted scatterplot smoothing; Lymph%, lymphocyte percentage; PA, prealbumin; SHAP, SHapley Additive exPlanations.

Supplementary Figure S6. SHAP waterfall plots illustrating individual risk attribution. Red arrows denote features that increase the predicted probability of acute postoperative protein depletion, whereas blue arrows denote features that decrease the probability.

(A) Risk attribution for a representative high-risk patient.

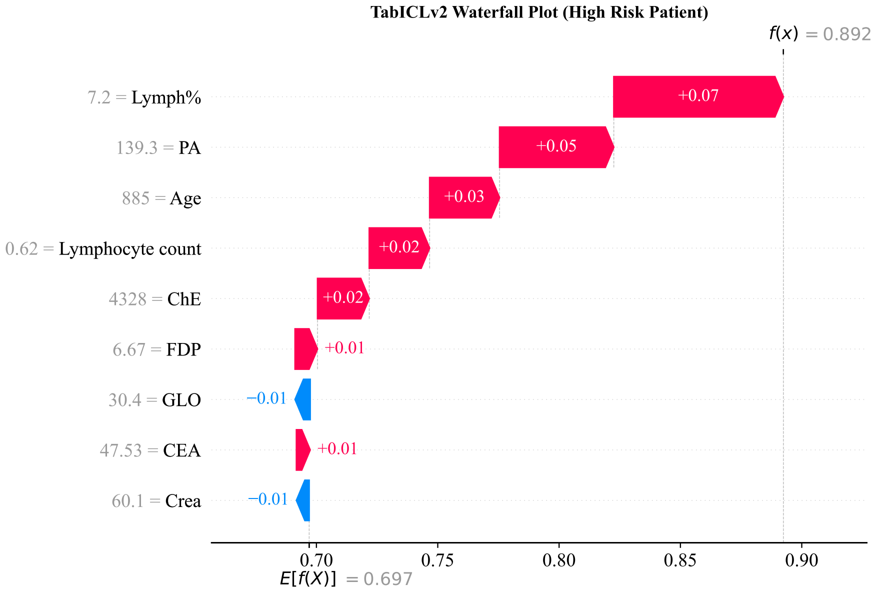

(B) Risk attribution for a representative low-risk patient.

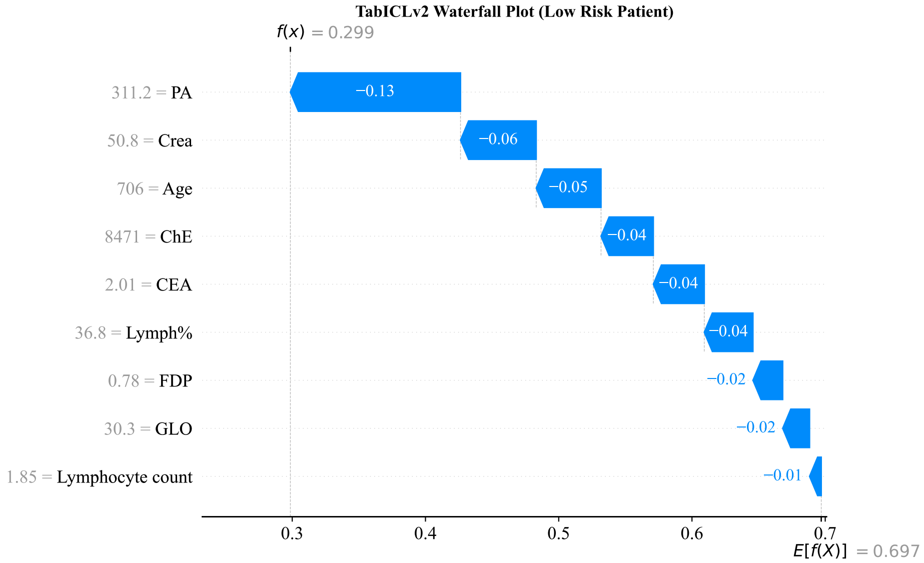

Supplementary Figure S7. User interface of the TabICLv2-based web application. The figure illustrates a representative preoperative scenario. Based on the input clinical parameters, the platform computes an absolute predicted probability of acute postoperative protein depletion (93.04%) and concurrently generates a localised SHAP waterfall plot. The visual output displays the directional impact and magnitude of specific features (e.g., Lymph%, PA) contributing to the individual risk estimate.

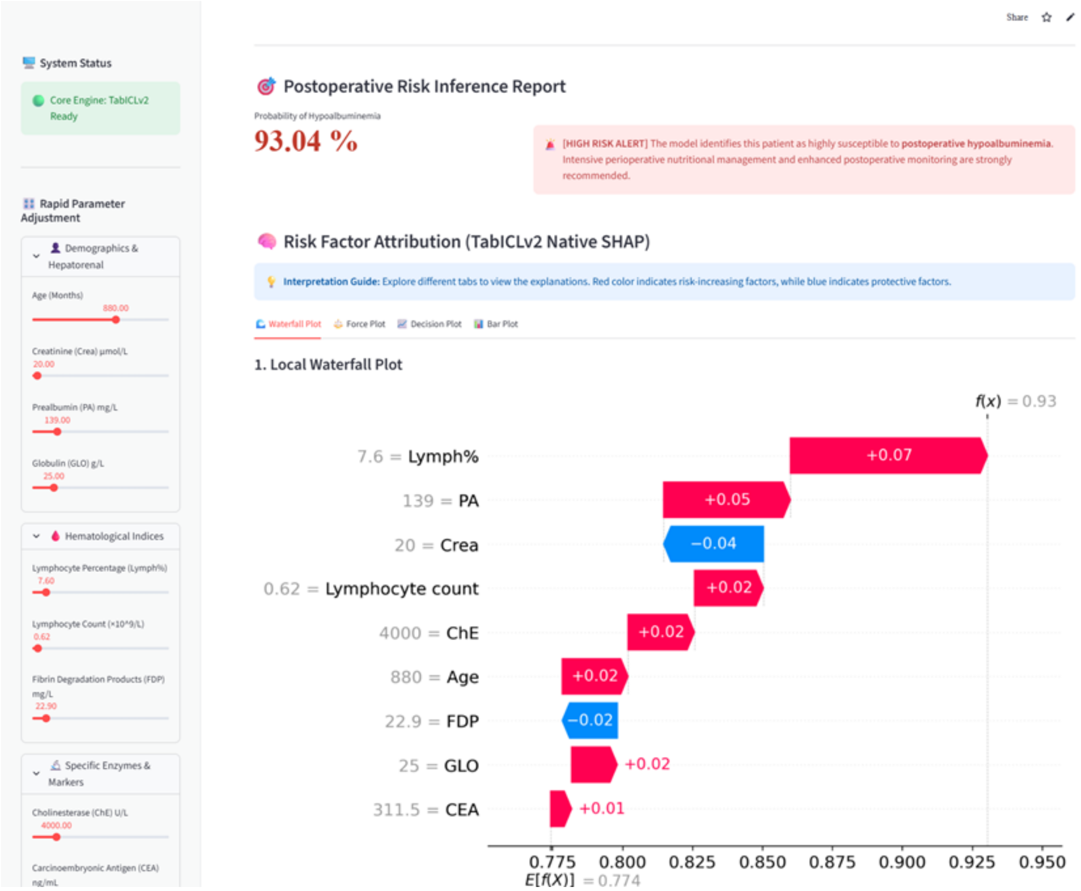

Supplementary Figure S8. Calibration curves for the TabICLv2 model using alternative outcome definitions. **(A)** Composite endpoint. **(B)** Albumin-only endpoint. **(C)** Total-protein-only endpoint. The x-axis represents the predicted probability, and the y-axis indicates the observed event frequency. The dashed diagonal line represents perfect calibration.

(A) Composite endpoint

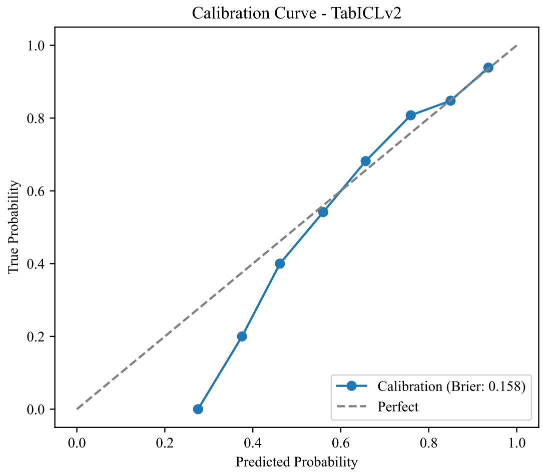

(B) Albumin-only endpoint

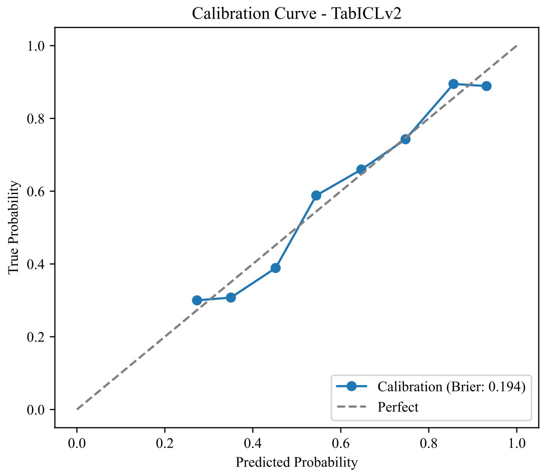

Supplementary Figure S8. Continued.

(C) Total-protein-only endpoint

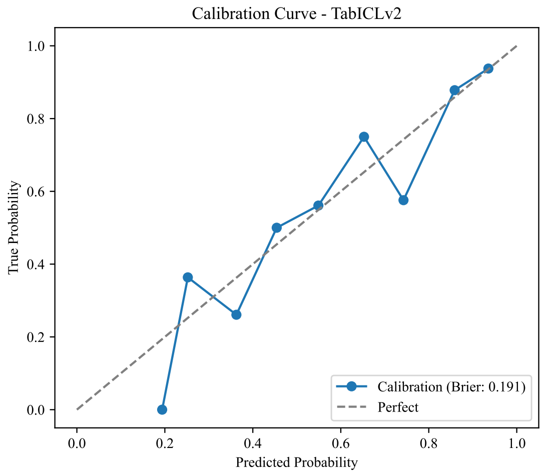

Abbreviations: ALB, albumin; TP, total protein; TabICLv2, Tabular In-Context Learning Version 2.

Supplementary Figure S9. Exploratory SHAP-based pairwise interaction visualisations for the TabICLv2 model. Each panel shows the SHAP value of the index feature on the y-axis and the corresponding observed feature value on the x-axis, with point colour representing the paired clinical variable. The black curve represents a LOWESS-smoothed trend, and the horizontal dashed line indicates a SHAP value of zero. Positive SHAP values indicate increased predicted probability of acute postoperative protein depletion, whereas negative SHAP values indicate decreased predicted probability. Age was displayed in years after conversion from the original month-based age variable. These plots were used for exploratory visual assessment of potential combined effects among core predictors rather than exact TreeSHAP interaction-value estimation.

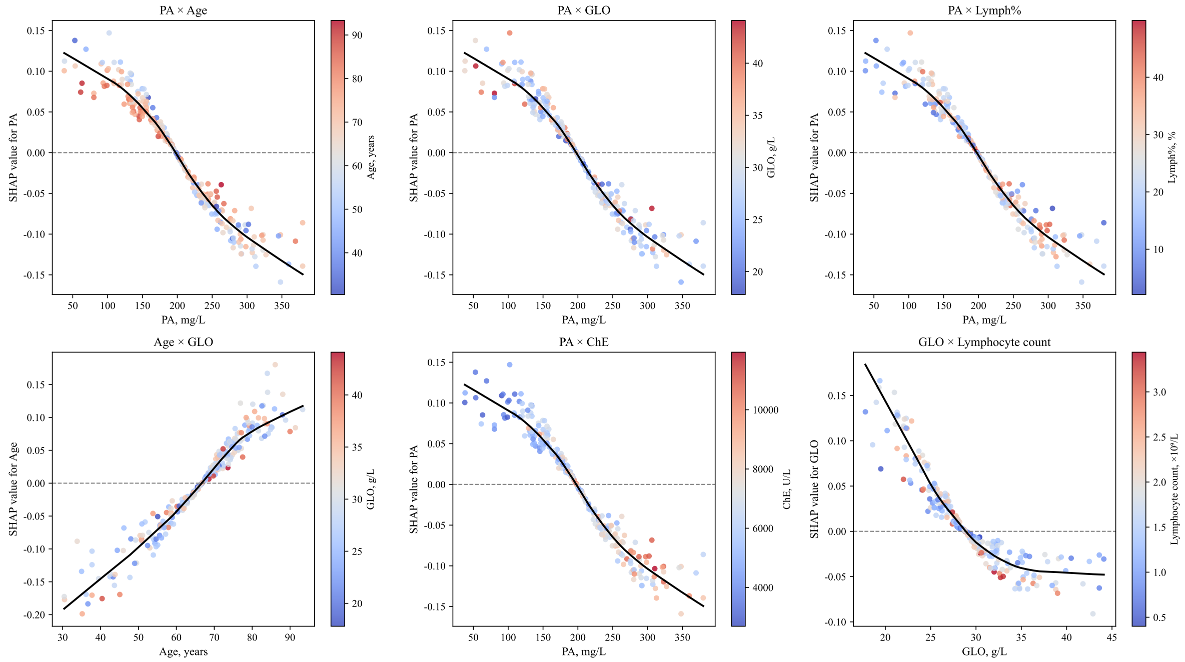

Abbreviations: SHAP, SHapley Additive exPlanations; LOWESS, Locally Weighted Scatterplot Smoothing; PA, prealbumin; GLO, globulin; ChE, cholinesterase; TabICLv2, Tabular In-Context Learning Version 2.
